# Mapping native and non-native vegetation in the Brazilian Cerrado using freely available satellite products

**DOI:** 10.1038/s41598-022-05332-6

**Published:** 2022-01-28

**Authors:** Kennedy Lewis, Fernanda de V. Barros, Marcio B. Cure, Christian A. Davies, Mariana N. Furtado, Timothy C. Hill, Marina Hirota, Demétrius L. Martins, Guilherme G. Mazzochini, Edward T. A. Mitchard, Cássia B. R. Munhoz, Rafael S. Oliveira, Alexandre B. Sampaio, Nicholas A. Saraiva, Isabel B. Schmidt, Lucy Rowland

**Affiliations:** 1 College of Life and Environmental Sciences, University of Exeter, Exeter, UK; 2 Department of Ecology and Zoology, Universidade Federal de Santa Catarina (UFSC), Florianópolis, Brazil; 3 Shell International Exploration and Production Inc, Shell Technology Centre, Houston, TX USA; 4 Department of Plant Biology, Institute of Biology, Universidade Estadual de Campinas (UNICAMP), Campinas, Brazil; 5 Department of Physics, Universidade Federal de Santa Catarina (UFSC), Florianópolis, Brazil; 6 School of GeoSciences, University of Edinburgh, Edinburgh, UK; 7 Department of Botany, Universidade de Brasília, Brasília, Brazil; 8 Centro Nacional de Avaliação da Biodiversidade e de Pesquisa e Conservação do Cerrado CBC Instituto Chico Mendes de Conservação da Biodiversidade - ICMBio, Brasília, Brazil; 9 Fundação Oswaldo Cruz, Rio de Janeiro, Brazil; 10 Department of Ecology, Universidade de Brasília, Brasília, Brazil

**Keywords:** Ecosystem ecology, Grassland ecology, Tropical ecology

## Abstract

Native vegetation across the Brazilian Cerrado is highly heterogeneous and biodiverse and provides important ecosystem services, including carbon and water balance regulation, however, land-use changes have been extensive. Conservation and restoration of native vegetation is essential and could be facilitated by detailed landcover maps. Here, across a large case study region in Goiás State, Brazil (1.1 Mha), we produced physiognomy level maps of native vegetation (n = 8) and other landcover types (n = 5). Seven different classification schemes using different combinations of input satellite imagery were used, with a Random Forest classifier and 2-stage approach implemented within Google Earth Engine. Overall classification accuracies ranged from 88.6–92.6% for native and non-native vegetation at the formation level (stage-1), and 70.7–77.9% for native vegetation at the physiognomy level (stage-2), across the seven different classifications schemes. The differences in classification accuracy resulting from varying the input imagery combination and quality control procedures used were small. However, a combination of seasonal Sentinel-1 (C-band synthetic aperture radar) and Sentinel-2 (surface reflectance) imagery resulted in the most accurate classification at a spatial resolution of 20 m. Classification accuracies when using Landsat-8 imagery were marginally lower, but still reasonable. Quality control procedures that account for vegetation burning when selecting vegetation reference data may also improve classification accuracy for some native vegetation types. Detailed landcover maps, produced using freely available satellite imagery and upscalable techniques, will be important tools for understanding vegetation functioning at the landscape scale and for implementing restoration projects.

## Introduction

Seasonally dry regions cover a large proportion of tropical land area^1^ and the importance of vegetation in these regions for carbon and water balance regulation and climate change mitigation is increasingly being recognised^2–4^. Generally, in seasonally dry tropical regions, complex underlying geology, combined with strong seasonal water-deficits and contrasting wildfire regimes generate a very heterogeneous vegetation cover spanning a range of different vegetation types, each thriving in different environmental niches^5–8^. Mapping the spatial distribution of different native and non-native vegetation types in seasonally dry tropical regions is key to understanding their functioning and threat from landcover change^9^. However, across large areas, this mapping is only viable using remotely sensed imagery^10^. Advances in remote sensing techniques are increasingly allowing us to identify vegetation characteristics at finer ecological scales^11–13^. However, few studies have focused on utilising recently available imagery and new remote sensing techniques to assist in identifying and quantifying the complexity of vegetation types across seasonally dry tropical regions.

The Brazilian Cerrado is a tropical savanna dominated biome, with marked wet and dry seasons, covering ~ 200 Mha^14^. Native vegetation spans a variety of distinct physiognomies (vegetation communities with distinct structures, height, spacing, dominant species, functional traits, phenology, soil or geological characteristics) which can be grouped into broad vegetation formation categories (grassland, savanna and forest)^15–17^ (Table 1). Vegetation can range from waterlogged grassland physiognomies (*campo limpo úmido*) to seasonally deciduous forest (*mata seca*) and xeromorphic woodland (*cerradão*) physiognomies across a small section of the landscape, often transitioning over spatial scales far smaller than the best existing global vegetation cover maps^18,19^, (e.g. 0.5–10 ha, Table 1). The distribution and coexistence of these vegetation types is broadly driven by changes in soil nutrients^20–22^, water availability^23–25^, geomorphology^26,27^ and vegetation-fire dynamics^28–30^. However, the interactions between and relative importance of factors in determining the distribution of different vegetation types, particularly for physiognomies that are transitional in nature, remains disputed^31,33^. Despite this complexity, native vegetation in most Cerrado land cover maps is broken down into broad formation level categories, masking the diverse nature and functioning of the multiple contrasting physiognomies within each of these classes (Table 1)^34–36^.

**Table 1:** Cerrado sensu lato physiognomies.

Formation	Physiognomy	Abbr	Characteristics	Canopy height (m)	Canopy cover (%)
Grassland (GRA)	Campo limpo úmido* *(open wet grassland)*	CLÚ	Annually to seasonally waterlogged soils, continual grasses, herbaceous and subshrub plants, without woody vegetative cover.	-	-
	Campo seco* *(grassland, well drained)*	CS	Well drained soils, continual grasses, herbaceous and subshrub plants, sometimes with sparse shrubby vegetation cover *(campo limpo seco, campo sujo)*.	-	< 5
	Campo rupestres* *(rupestrian grassland)*	CAMR	Semi-continual grasses and herbaceous species with rocky outcrops of quartzite, sandstone or ironstone (canga) sometimes in montane systems or at high elevations, with some low stature trees and shrubs usually on well drained soils.	-	< 5
Savanna (SAV)	Cerrado sensu stricto* *(typical cerrado)*	CSS	Continual grassy, subshrub sublayer with xeromorphic evergreen to semi-deciduous tree cover at various densities on well drained soils.	3–6	10–60
	Cerrado rupestre* (rupestrian cerrado)	CERR	Continual to sparse grass sublayer, with low density tree cover on well drained soils with rocky outcrops.	2–3	5–10
	Vereda* *(palm swamp)*	V	Annually to seasonally waterlogged soils, continual grasses, herbaceous and subshrub plants with localised stands of *Mauritia flexuosa* L. f. (buriti palms) in waterlogged depressions or in close proximity to gallery forests.	12–15 (palms)	-
Forest (and woodland) (FOR)	Cerradão* *(dense cerrado woodland)*	C	Dense xeromorphic trees with scarce herbaceous cover, tree species composition is floristically similar to *cerrado *sensu stricto.	8–15	60–90
	Mata ciliar* *(riparian forest)*	-	Semi-deciduous to evergreen forest alongside watercourses, typically extending less than 100 m from the water edge.	8–12	60–90
	Mata de galeria* *(gallery forest)*	MG	Semi-deciduous to evergreen forest forming closed corridors along narrow watercourses.	20–30	70–95
	Mata seca *(seasonally dry tropical forest)*	-	Seasonally deciduous and semi-deciduous forests formed on well drained nutrient rich soils.	15–25	70–95

Adapted from Eiten (1978)^15^, Oliveira and Marquis (2002)^17^ and Ribeiro and Walker (2008)^16^. The eight native physiognomies present across the study area indicated*. N.B. *Mata Ciliar* and *Mata de Galeria* are grouped to form a single class.

Understanding these distinctions at scale is essential to large-scale conservation planning, particularly within biomes such as the Cerrado^37–39^. As a result of extensive land use changes (LUC) only ~ 88 Mha (46%) of native Cerrado vegetation cover now remains, with as little as 20% left undisturbed and less than 7% within Protected Areas^40,41^. Detailed vegetation maps are essential to aid in identifying priority physiognomies and areas to conserve in this biome. Furthermore, physiognomy level maps would be valuable for guiding field sampling strategies when assessing plant function and biodiversity and for more detailed landscape-scale process modelling^42,43^. Perhaps most urgently, physiognomy level maps would facilitate large scale restoration across this biome, which is key to recovering many of the ecosystem services already lost through unsustainable land management practices^40,44^.

The potential for restoration of native vegetation in the Cerrado is high^40^. In addition to private and international stakeholders^45,46^, the Brazilian government have pledged to restore 2.1 Mha of degraded land in the Cerrado to native type vegetation by 2030^47^. However, restoration in the Cerrado remains challenging. As a result of land-use changes, areas targeted for restoration have often undergone significant changes in soil structure and nutrient composition^48,49^. Despite this, neighbouring native vegetation is a good indicator of whether passive or active restoration approaches might be suitable^44^, the type of vegetation to restore a degraded area to^50^, prospective propagule availability^51^ and the potential for maximising ecosystem connectivity^52^. Understanding the matrix of vegetation surrounding a potential restoration area might also minimize the risk of restoration failure due to invasion of exotic species (particularly pasture grasses)^53^ and improve our ability to evaluate the potential fire risk to newly restored vegetation.

Producing detailed maps of Cerrado physiognomies to inform conservation and restoration is challenging. Different vegetation types may initially appear very similar in the field, and are hard to distinguish in satellite imagery even at a high spatial resolution^16,54^, however many are floristically distinct^16,55,56^ and have markedly different soil properties and phenological responses to water deficit^57–59^. The MapBiomas project produces annual maps of native vegetation at the formation level (forest, savanna, grassland) and non-native vegetation across Brazil at a 30 m spatial resolution from 1985 onwards^60^. The project leverages the Google Earth Engine (GEE) platform; image processing standard algorithms, cloud computing capabilities and freely available Landsat imagery within the GEE repository^61,62^. These maps have been invaluable for vegetation degradation and deforestation monitoring^34,61^, assessing future LUC and carbon storage^63,64^, and understanding ecosystem functionality^65^. Recently, several smaller scale case studies have successfully detected and mapped Cerrado vegetative types at the physiognomy level. These studies have used various combinations of surface reflectance imagery to assess vegetation reflectance spectra and as a proxy for canopy structure and photosynthetic activity^66–69^. In addition, some have also used synthetic aperture radar (SAR) products, which provide valuable information on vegetation structural characteristics and soil and vegetation moisture^70–72^. Often, techniques that capture vegetation phenological patterns using imagery from multiple time periods are used^34,66,73^. Coarser scale, locally sampled soil properties and drainage datasets are sometimes utilized^67,74^. However, despite these examples of progress towards physiognomy-level vegetation identification, many do not use freely available input imagery or easily upscalable techniques and none incorporate both native and non-native landcover types in-situ.

Here, using a large case study area of 1.1 Mha we examine the potential to map Cerrado vegetation at the physiognomy level using seven different combinations of freely available SAR (Sentinel-1) and surface reflectance (Sentinel-2 or Landsat-8) remotely sensed products. The different combinations test the advantages of using the different products, but each follow the same 2-stage classification implemented in GEE. We consider vegetation burning when selecting ground reference data as a quality control step, and also explore the impact of incorporating seasonal metrics into the collection of input imagery.

## Method

Vegetation reference data was collected across the study site, quality control procedures were then applied to reduce the impact of vegetation burning on classification accuracies. Seven landcover maps were then generated at either the vegetation formation or physiognomy level with a spatial resolution of either 20 m, the nominal resolution of most Sentinel-2 bands, or 30 m, the resolution of Landsat-8. Each of the seven formation and physiognomy maps were generated using different feature spaces. A feature space refers to the collection of feature vectors used to characterise a dataset. In this instance, each map pixel represents an area of land covered in vegetation (the dataset), and various combinations of input satellite images (the vectors) are used to describe the vegetation and generate an output map.

### Study site

**Figure 1:**
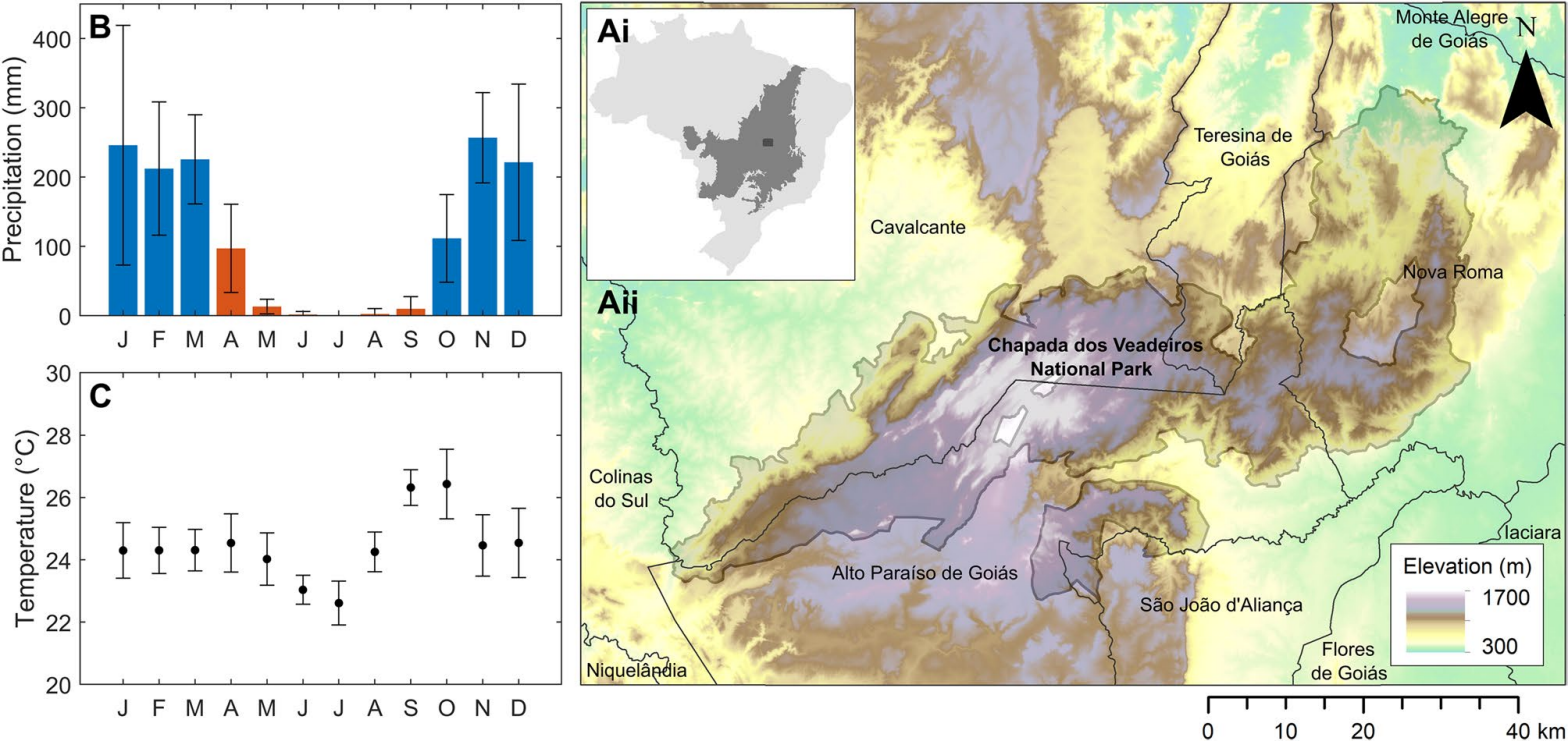
Study area, location, and climate. **Ai.** Study area location within Central Brazil, biome boundaries indicated (IBGE, 2019). **Aii.** Study area and CVNP extent, elevation taken from the COP-GLO-30 DEM. Mean total monthly total precipitation (mm) **(B)** and mean monthly temperature (°C) **(C)** across the CVNP, taken from the ERA5 gridded climate reanalysis at a 0.25 arc degree grid cell. Monthly values presented are 10-year averages from 2009 till 2019 (error bars indicate standard deviation from the CVNP average over the 10 year period). Monthly precipitation totals < 100 mm are indicated in red.

The study area (~ 1.1 Mha) is located in Goiás State, Brazil (Fig. 1). The climate is typical of the central Cerrado region; wet season rainfall is followed by intense water deficit in the dry season (Fig. 1B, C). Total annual precipitation at the site is ~ 1400 mm, with ~ 130 mm of this falling in the dry season (April to September) (mean 2009–2019, Fig. 1B)^75^.

Situated within the study area is the Chapada dos Veadeiros National Park (CVNP) a legal reserve, covered by native vegetation with an area of ~ 240,600 ha (Fig. 1)^76,77^. Across the park, at least eight well preserved Cerrado physiognomies are thought to be present (Table 1). As a consequence of alterations to the boundaries of the park over time, several private properties (and hence non-native land cover types) are also situated within the CVNP limits. The area surrounding the CVNP is mosaic of native vegetation, which has undergone various levels of degradation, and other land uses, including large scale and smallholder planted pastures and agriculture (Table 2). Soils can be well-drained to annually saturated and are predominantly Cambissolos, Plintossolos, Gleysols and Neossolos; however, both the CVNP and the surrounding area are characterised by a high pedological diversity and topographic and geological complexity (Fig. 1Aii)^78,79^. This results in a high level of local-scale vegetation heterogeneity within the same climatic envelope.

**Table 2:** Non-native vegetation and landcovers present across the study site.

Formation	Abbr	Characteristics
Pasture	PAST	Active or abandoned planted pastures mostly dominated by planted African grass species (*Brachiaria*)
Plantation Forest	PF	Monoculture forest plantations (mostly *Eucalyptus* plantations across the study site)
Agriculture	A	All annual and perennial agricultural land uses
Water	W	Any open waterbody larger than 20 m^2^
Non-vegetated	NV	Non-vegetated soil, rock or urban areas

### Vegetation ground reference data

We gathered all available vegetation type reference data across the study site between 2017 and 2020. Using expert knowledge, collating data from multiple studies and surveys, 1686 unique ground reference sites (GPS coordinates) were identified across the eight native vegetation physiognomies (Fig. S1). Non-native vegetation types were also identified in the field, with further GPS coordinates selected following the inspection of timeseries imagery in Google Earth (a combination of satellite imagery and arial photos) and the MapBiomas Collection 5 land cover map for 2018^34,60,61^.

To increase the number of pixels available to train and validate the classifier, reference zones spanning multiple pixels were sampled at each unique reference site. For grassland physiognomies, reference zones were manually delineated around a GPS location to select vegetation reference pixels, informed by field visits and high spatial resolution imagery in Google Earth. For the majority of savanna and forest physiognomies, sample buffer zones with a radius of between 20 and 40 m were applied (depending on the area and consistency of vegetation cover). Based on observations in the field, for the vereda class, a smaller buffer radius of 12 m was applied to ensure reference sites were centred on palm trees, in most cases sampling a single pixel at each location. Following the same logic, a 12 m buffer zone was also used for the *mata de galeria* class.

After burned area masking (see next section) a total of 6603 pixels (at a 20 m spatial resolution) across the 1686 sites were sampled for the eight native vegetative physiognomies (Fig. S1, Table S1).

### Burned reference data masking

Managed, accidental and natural fires are common across the study area. In order to minimise the effect of burned vegetation contaminating classification accuracies, a global burned area product was selected and used to disregard any vegetation reference areas, identified in the field between 2017 and 2020, that may have burned within the input imagery period (October 2018 to September 2019).

Before undertaking this quality control procedure, the accuracy of two global burned area (BA) products was evaluated following the methodology of Rodrigues et al.^80^ (Supplementary Sect. 1). Commission errors (CE, the fraction of unburned pixels mistakenly classified as burned) and omission errors (OE, the fraction of burned pixels not detected by the product) relative to a Cerrado specific reference BA product were assessed (Supplementary Sect. 1).

In all further analysis, the MODIS MCD64A1 v6 BA product^81^ was then used to assess burned area extent, produce a burned area mask and disregard any burned vegetation reference areas.

### Input imagery and feature space

#### Imagery selection and pre-processing

All imagery used, aside from elevation data, was taken from within the GEE repository^82^. To increase the probability of finding a cloud free pixel across all areas (for surface reflectance imagery), all available imagery from October 2018 to September 2019 for each product within the repository was considered.

Ground Range Detected (GRD) Sentinel-1 (S1) C-band SAR imagery was acquired at a 10 × 10 m pixel size, in Interferometric Wide swath (IW) mode, on the descending pass. Images were ground- range detected and log scaled (σ^0^dB) upon acquisition, in the VH (vertical transmit, horizontal receive) and VV (vertical transmit, vertical receive) polarisation. The VH to VV ratio was also calculated, as this has provided additional power when monitoring vegetation structure and vegetation and soil moisture in other studies^83,84^. C-band backscatter values were transformed to σ^0^ prior to ratio calculation. S1 images were resampled (nearest neighbour) to match the final feature space spatial resolution for use alongside either S2 or L8 data (20 or 30 m) and a per pixel mean calculated across the image collection for the time period considered.

The effectiveness of using Sentinel-2 (S2) or Landsat-8 (L8) surface reflectance imagery to identify Cerrado vegetation types was assessed separately at the study site. S2 Level-2A surface reflectance imagery was filtered to include only scenes with a cloud cover of < 70%. A cloud and dark pixel mask based on the S2 ESA Scene Classification^85^ was then applied, followed by a second masking procedure using a built-in cloud and shadow bit mask^86^. Similarly, L8 surface reflectance imagery was also acquired, filtered, and a cloud and shadow bit mask applied^87^. In addition to surface reflectance bands (Table 3), for all S2 and L8 scenes acquired across the study site area, several vegetation indices were calculated (NDVI, EVI2, SAVI, SWIR21, Table 3). These indices were chosen as they are a good proxy for vegetation productivity or woody vegetation coverage and have been successfully used in other studies to characterise Cerrado vegetation^34,88–90^. Where required, all bands and indices were resampled from their initial spatial resolution to a resolution of 20 m (S2) or 30 m (L8) and a per pixel median was calculated across the period considered. The final images were visually inspected for any remaining cloud presence.

**Table 3:** Input feature layers produced for the seven alternative classification feature spaces.

Feature layer	Wavelength/formula/polarisation S2 (L8)	S2/L8 equivalent	Initial spatial resolution(m) S2 (L8)	Image season/image statistics
**Surface reflectance from Sentinel-2 (S2) and Landsat-8 (L8)**				
*Reflectance bands*				
Blue (B)	492/492 nm (452–512 nm)	Y	10 (30)	Ann, Wet, Dry
Green (G)	560/559 nm (533–590 nm)	Y	10 (30)	Ann, Wet, Dry
Red (R)	665/665 nm (636–673 nm)	Y	10 (30)	Ann, Wet, Dry, A_var_
Red Edge 1 (R1)	704/704 nm	-	20	Ann, Wet, Dry, A_var_
Red Edge 2 (R2)	741/739 nm	-	20	Ann, Wet, Dry, A_var_
Red Edge 3 (R3)	783/780 nm	-	20	Ann, Wet, Dry, A_var_
NIR	833/833 nm (851–879 nm)	Y	10 (30)	Ann, Wet, Dry, A_var_
Red Edge 4 (R4)	865/864 nm	-	20	Ann, Wet, Dry, A_var_
SWIR 1	1614/1610 nm (1566–1651 nm)	Y	20 (30)	Ann, Wet, Dry
SWIR 2	2202/2186 nm (2107–2294 nm)	Y	20 (30)	Ann, Wet, Dry
*Vegetation index*				
NDVI (normalized difference vegetation index)	$NDVI=\frac{NIR-red}{NIR+red}$	Y	10 (30)	Ann, Wet, Dry, A_var_, T_W_, T_D_
EVI2 (enhanced vegetation index 2)	$EVI=2.5*\frac{NIR-red}{NIR+2.4\left(red\right)+1}$	Y	10 (30)	Ann, Wet, Dry, A_var_, T_W_, T_D_
SAVI (soil adjusted vegetation index)	$SAVI=1.5*\frac{NIR-red}{NIR+red+0.5}$	Y	10 (30)	Ann, Wet, Dry, A_var_, T_W_, T_D_
SWIR21	$SWIR21= \frac{SWIR2}{SWIR1}$	Y	20 (30)	Ann, Wet, Dry, A_var_
**Sentinel-1**				
*Polarisation*				
σ^0^_VH_dB (VH)	VH (σ^0^dB)	n/a	20*	Ann, Wet, Dry, A_var_
σ^0^_VV_dB (VV)	VV (σ^0^dB)	n/a	20*	Ann, Wet, Dry, A_var_
*VH:VV Ratio*				
σ^0^_VH_VV_ (VH:VV)	$\frac{VH}{VV}$(σ^0^)	n/a	20*	Ann, Wet, Dry
**COP-DEM GLO-30**				
Slope	Slope (˚)	n/a	30	-

Vegetation indices are taken from Alencar et al. (2020)^34^, Xue and Su (2017)^88^, Parente and Ferreira (2018)^89^ and Hill et al.^90^. The study area is situated within a single scene for Sentinel-1, mosaic images (2 scenes) were produced for Sentinel-2 (S2) and Landsat-8 (L8) derived features. Bands and vegetation indices, available or derived from both the S2 and L8 products are indicated (S2/L8 Equivalent). Annual (Ann, 01/10/18 to 30/09/19), wet season (Wet, 01/10/2018–31/03/2019) and dry season (Dry, 01/04/2019–30/09/2019) images were produced for some feature layers. Annual variance (A_var_), wet and dry season texture images (T_W_, T_D_) were also produced. *Pixel size 10 × 10 m (S1). See Fig. S2 to S5.

Three time periods were considered for the bands, polarisations and derived vegetation indices or ratios taken from each input product (S1, S2 and L8) in addition to other image statistics (Table 3). Given the strong constraint rainfall seasonality and temperature may have on vegetation phenology (Fig. 1B, C), to assess the importance of incorporating phenological characteristics into the input imagery feature space, annual, wet season and dry season images were produced. Wet and dry season months were defined using the ERA5 gridded climate reanalysis dataset within GEE (dry season months = monthly total rainfall < 100 mm, 10-year average across the study site)^75^ (Fig. 1B, C). As the magnitude of plant phenological responses observed in some bands and vegetation indices is expected to be larger for some vegetation types, the per pixel variance over the annual imagery collection (A_var_) was also calculated for some products (Table 3). When visually inspected, good alignment was observed between all imagery products, however, this may require correction if applied in other regions.

Texture layers for both wet and dry season imagery (T_W_, T_D_) were also produced for some S2 layers by taking the standard deviation of pixels within a 1 ha moving window^91^. These texture images were incorporated to assess the variability of a signal amongst a pixel and its neighbours, as some physiognomies form distinctive linear patterns in the landscape (*mata de galeria, vereda*)^91–93^.

Elevation data was taken from the COP-GLO-30 DEM^94^, and terrain slope was then calculated. This slope layer was included in all classification feature spaces, as different vegetation types are typically located in different topographic zones.

#### Alternative feature spaces

The potential for mapping Cerrado vegetation at the formation and physiognomy levels was assessed for seven different input feature spaces, each comprised of a unique input imagery combination (Table 4, Table S2). Pairwise comparisons of the output vegetation maps and map accuracies resulting from certain feature spaces (Table 4, ‘Difference’) can be used to assess the impact of adding or removing imagery on the accuracy of the classification.

**Table 4:**
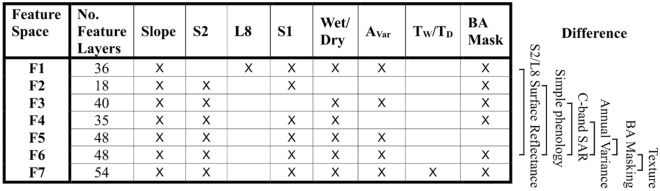
Alternative feature spaces and input feature layers.

Summary of feature layers for each of the seven alternative feature spaces, full layers listed in Table S2. The difference between Feature Space 6 (F6) and each other alternative feature space is indicated.

Alternative feature spaces were made up of seasonal multispectral imagery from L8 rather than S2 (F1), exclusively annual S1 and S2 imagery (F2), seasonal multispectral S2 imagery only (F3), seasonal S2 and S1 imagery without A_var_ imagery (F4), seasonal S2 and S1 imagery with A_var_ imagery (F6) and further incorporation of texture layers (F7). A further classification was run using F6 imagery without applying burned reference area masking as a quality control step, hereby referred to as F5.

### Classification routine

A 2-stage classification routine using a Random Forest (RF) classifier was implemented in GEE^95^. Initially pixels were assigned a stage-1 land cover type, where native vegetation is classified to the formation level (herbaceous, savanna or forest), alongside non-native covers (Fig. S7). At the second stage, native vegetation pixels were re-classified to the physiognomy level within their assigned formation class. The process was repeated with different training/validation splits of the ground reference data, vegetation reference pixels were split according to unique zone (rather than sampling pixels directly) to reduce spatial autocorrelation and the overestimation of map accuracies^96^. Random training/validation splits were standardized for each alternative feature space assessed. This 2-stage classification routine was implemented to optimise the use of available training data and allow the production of both a simple (formation level) and more complex (physiognomy level) map output. In addition to this, a 2-stage classification allows the re-training and optimisation of the Random Forest classifier at the second stage to distinguish between a smaller group of more similar vegetative classes at the physiognomy level.

At the initial stage of the classification process, a validation set of the ground reference data (20% of each class) was withheld to be used for the calculation of accuracy metrics at stage-2. After this, the remaining ground reference data was again randomly split and 80% of the remaining data was then used to train the RF classifier at stage-1 and stage-2. The classification was repeated 50 times at each stage using different subsets of the remaining ground reference data. For each classification, a RF classifier with 100 trees was used (RF parameters were not tuned here, however, tuning may improve predictive performance in further analyses). At the end of each stage, the per pixel output class of the 50 repeat runs was compared and a final formation (stage-1) or physiognomy (stage-2) selected if a majority output class (> 50%) was reached (Fig. S7). At stage-1 pixels where a majority was not reached remained unclassified; at stage-2 pixels where a physiognomy level classification cannot be reached remained classified to the formation level. The entire process was then repeated 10 times with a different validation set (20%) of ground reference data withheld at the initial stage, resulting in 500 potential map outputs per feature space assessed.

### Distance based reclassification of vereda pixels

Following the classification routine, to reduce commission errors for the vereda class (savanna formation), a further vereda specific distance-based step based on landscape characteristics was added. During ground reference data selection, we centred vereda reference data on palm pixels rather than on the surrounding waterlogged herbaceous layer (Table 1). Therefore, the herbaceous layer around vereda palms in this study was typically classified as *campo limpo úmido*. Both physiognomies have waterlogged soils for much of the year, and despite some differences, their herbaceous layers are floristically similar^55^. To reduce commission errors for the vereda class, which were evident after classification stages 1 and 2, vereda pixels were re-classified based on their proximity to pixels assigned to the *campo limpo úmido* class (see Fig. S8). For each potential map output (n = 500), if a vereda pixel is > 1 ha from a *campo limpo úmido* pixel, the vereda pixel was assumed to be misclassified. After detection the erroneous pixel was re-classified as the most common neighbouring savanna or forest physiognomic vegetation type (*cerrado *sensu stricto*, cerrado rupestre, cerradão* or *mata de galeria*), within a 1 ha moving window centred on the vereda pixel. A distance of 100 m was chosen based on field observations and inspection of imagery in Google Earth.

### Error assessment and classification probability

For each unique classification run (n = 500, for seven alternative feature spaces), overall map accuracy (OA, accounting for all misclassifications across all classes), and per-class user’s (UA, accounting for commission errors, the fraction of other classes mistakenly classified as a class in the final map) and producer’s (PA, accounting for omission errors, the fraction a class mistakenly classified as another class in the final map) accuracies were calculated. Accuracies at stage-2 were calculated using the ground refence data withheld at the start of the classification process, considering misclassification at both stages. At stage-1 the remaining ground reference data that has not been used to train the classifier after the second dataset split was used. Therefore, in both cases, independent test data (not used to train the models) was used to derive these figures. Output maps were also visually inspected with comparison to in-situ imagery in Google Earth.

The median and interquartile range (IQR, here, the difference between Upper = 75th percentile and, Lower = 25th percentile) of OA, UA and PA across the 500 unique classification repeats are presented and discussed. To assess the significance of increases or decreases in map accuracy observed when the input feature layers are varied, sample means were compared using one-way analysis of variance (ANOVA, samples were normally distributed, with constant variance when visually inspected). Subsequent post-hoc pairwise comparisons (Tukey Multiple Comparison tests) were then applied and the results of these pairwise comparisons are outlined in the results section. The per pixel classification probability was then calculated as the percentage of times each pixel was classified as its final output class at each stage based on different training datasets. The estimated area of each landcover type across the study site (taking into account per class accuracies) and 95% CI was assessed following the methodology of Olofsson et al.^97^ for each unique classification.

## Results

The output maps and accuracies from the results of seven unique classifications, each with a distinct feature space are presented. The extent of vegetation burning events across the CVNP region, the amount of ground reference data disregarded due to potential burning, and an assessment of BA product accuracies is initially outlined. Vegetation maps were then produced at the formation level (herbaceous, savanna and forest) and at the physiognomy level, where there are eight unique classes. Classification accuracies; overall, users and producers accuracies, are presented for each feature space at each classification level. Finally, the landcover map with the highest accuracy is presented alongside the final per class output probabilities and areas.

### Burned area across the CVNP region

The accuracy of both global BA products assessed was low at the study site, however, the MCD64A1_v6 product showed slightly better agreement with the Cerrado specific reference BA product (Supplementary Sect. 1, Fig. S6). The MCD64A1_v6 product and was used for all further analysis and the quality control of input imagery.

Over the feature layer imagery collection period the mean CE and OE of the MCD64A1_v6 product was 30.2 ± 10.9% and 48.4 ± 23.7% respectively, with a mean bias of 0.77 ± 0.36 across the study site. Omission errors over the feature layer imagery collection period reduced slightly (42.95 ± 23.6%) when only reference burn scars with an area > 0.5 km^2^ (the native spatial resolution of MCD64A1_v6) were included. Over the 3 years of vegetation reference data collection prior to and including the feature layer imagery collection period (2017–2019) CE and OE rose to 56.3 ± 26.6% and 70.6 ± 21.6% successively.

**Figure 2:**
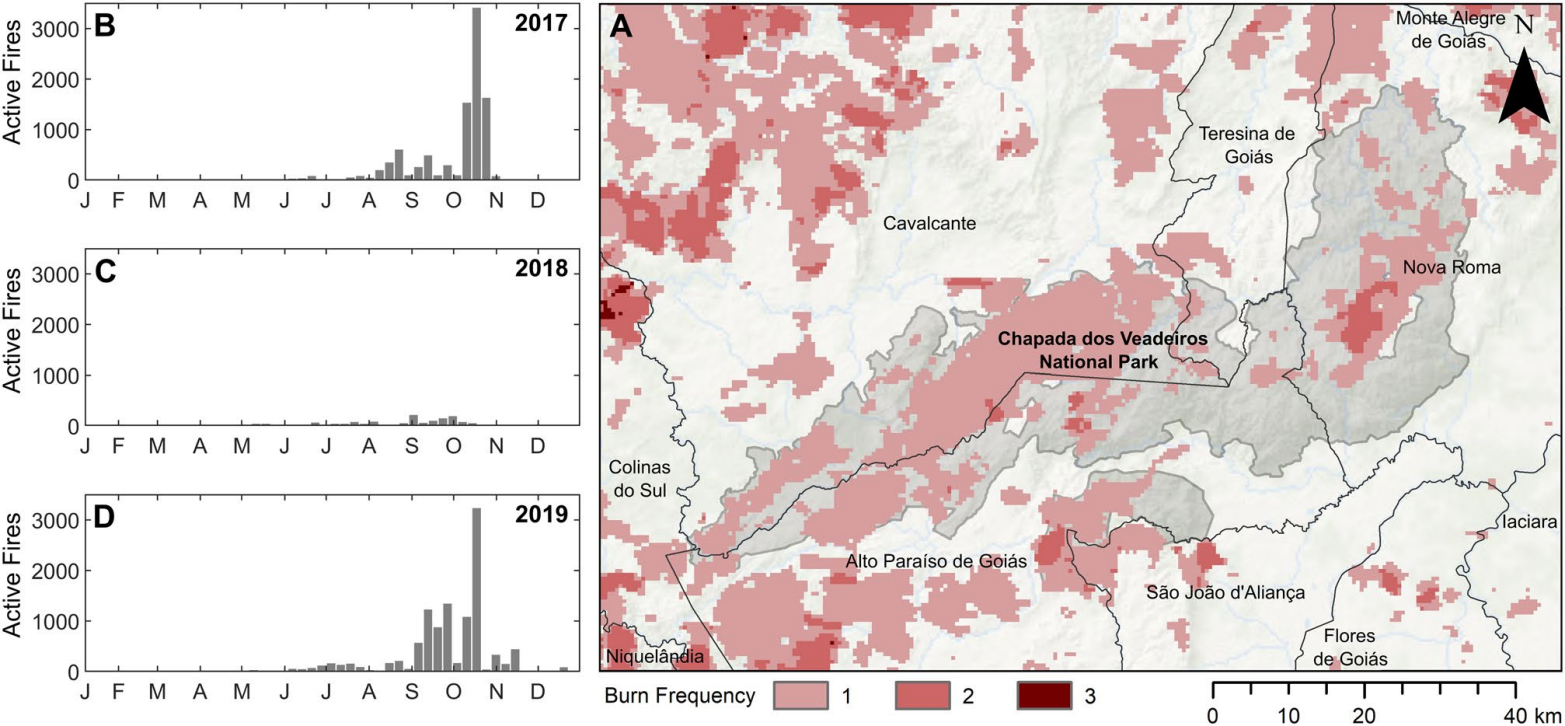
Total burned area and burn timing across the CVNP study area. Annual burn timing for 2017 (**A**), 2018 (**B**) and 2019 (**C**), daily fire hotspot count across the study area taken from the VIIRS 375-m active fires product. (**D)** Burned area frequency form Jan 2017 to Dec 2019, taken from the MCD64A1_v6 BA product.

Over this same period (Jan 2017–Dec 2019), 25.9% of the study area burned at least once, and 4.1% burned twice, as detected in the MCD64A1_v6 BA product (Fig. 2A). The majority of fires occurred in the late dry and early wet season (Fig. 2B–D). Following vegetation reference zone burned area masking as part of the quality control procedure, the number of available reference pixels reduced by 1.1%, 3.9% and 2.4% for herbaceous, savanna and forest physiognomies and by 0.7% for non-native vegetated classes.

### Classification accuracies

#### Overall accuracy

**Figure 3:**
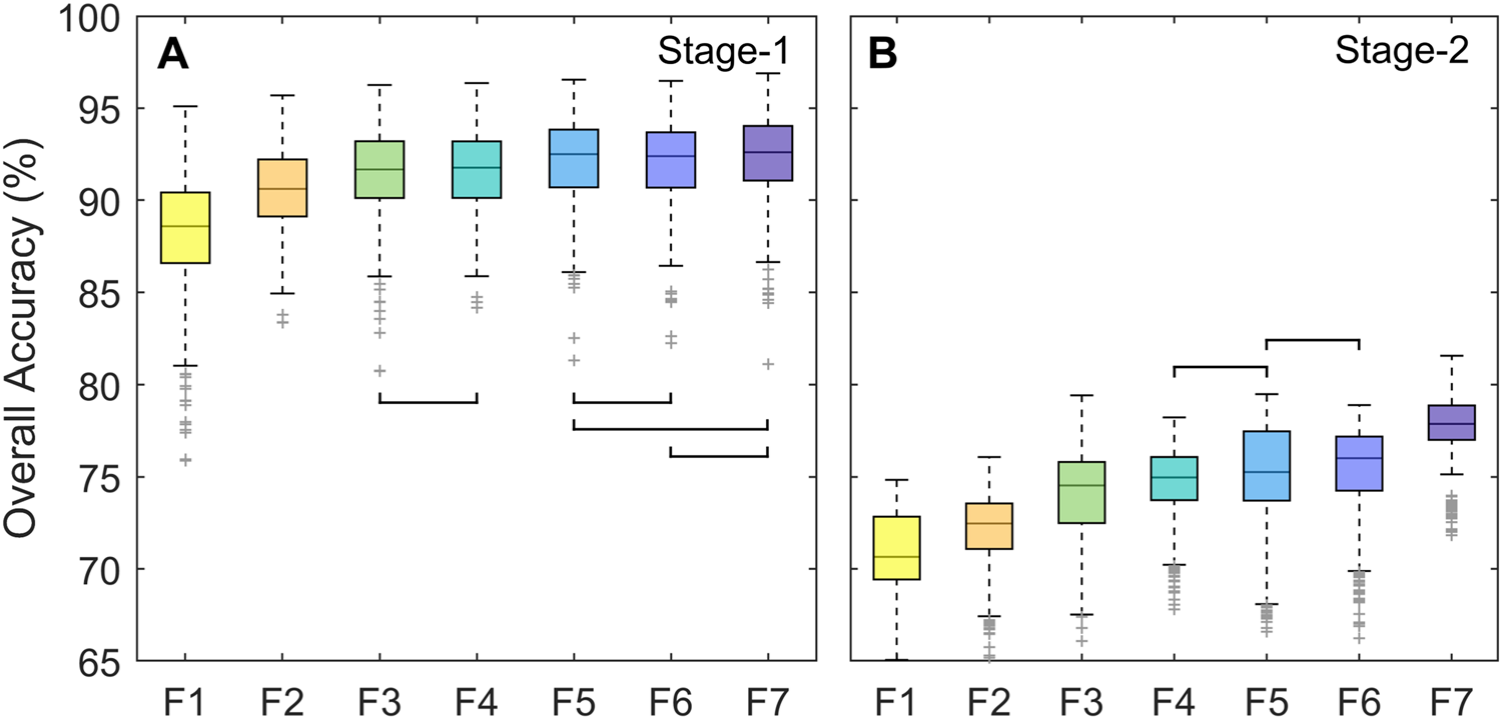
Overall map accuracies at classification stages 1 and 2. (**A)** Stage-1 formation level map OA, including native and non-native vegetated and non-vegetated classes, for the seven alternative feature spaces F1–F7. (**B)** Stage-2 physiognomy level map OA for native vegetation classes only, across the seven alternative feature spaces F1–F7. For each feature space, the classification process has been repeated 500 times with different training/validation vegetation reference data splits. Aside from the paired groups indicated in black, all post-hoc pairwise comparisons between feature spaces (Tukey Multiple Comparison tests) where significant p ≤ 0.05.

Classification overall accuracies at the formation level (stage-1, forest, savanna and grassland, non-native vegetation, and non-vegetation classes) were high for all feature spaces compared (Fig. 3A). At stage-1 median OAs ranged from 88.6% for F1 to 92.6% for F7. The classification maps resulting from all input imagery combinations had a similar OA accuracy. However, a marginal but significant improvement in map OA was observed when a seasonal Sentinel-1 and Sentinel-2 feature space (F6) was used, when compared to the seasonal Sentinel-1 and Landsat-8 equivalent (F1) (p < 0.001). Adding texture imagery (F7) and undertaking the classification reference zone burned area masking (F5) did not significantly increase or reduce map OA when compared F6. In contrast, when compared to F6, removing Sentinel-1 C-band SAR (F3), removing annual variance imagery (F4) and using a fully annual feature space (F2) resulted in a significant reduction in OA (p ≤ 0.05).

At stage-2 OAs for the eight native physiognomy level classes were lower, ranging from 70.7% (F1) to 77.9% (F7) (Fig. 3B). Here, a larger variation in OA between the feature spaces was observed, albeit still relatively small. When compared, the map OA for F5 was not statistically different from F4 or F6 (p > 0.05), however, map OAs for all other feature spaces were statistically different from all other alternatives (p ≤ 0.01). At stage-2, incorporating seasonal imagery (Wet, Dry) and indicators (Avar) for all input layers (F2, F6) had a greater effect on map OA than the inclusion of seasonal SAR imagery (F3, F6). A small, non-significant increase in map OA was observed between F5 and F6, when reference zone burned area masking was applied (Fig. 3B).

#### Users and producers accuracy

**Figure 4:**
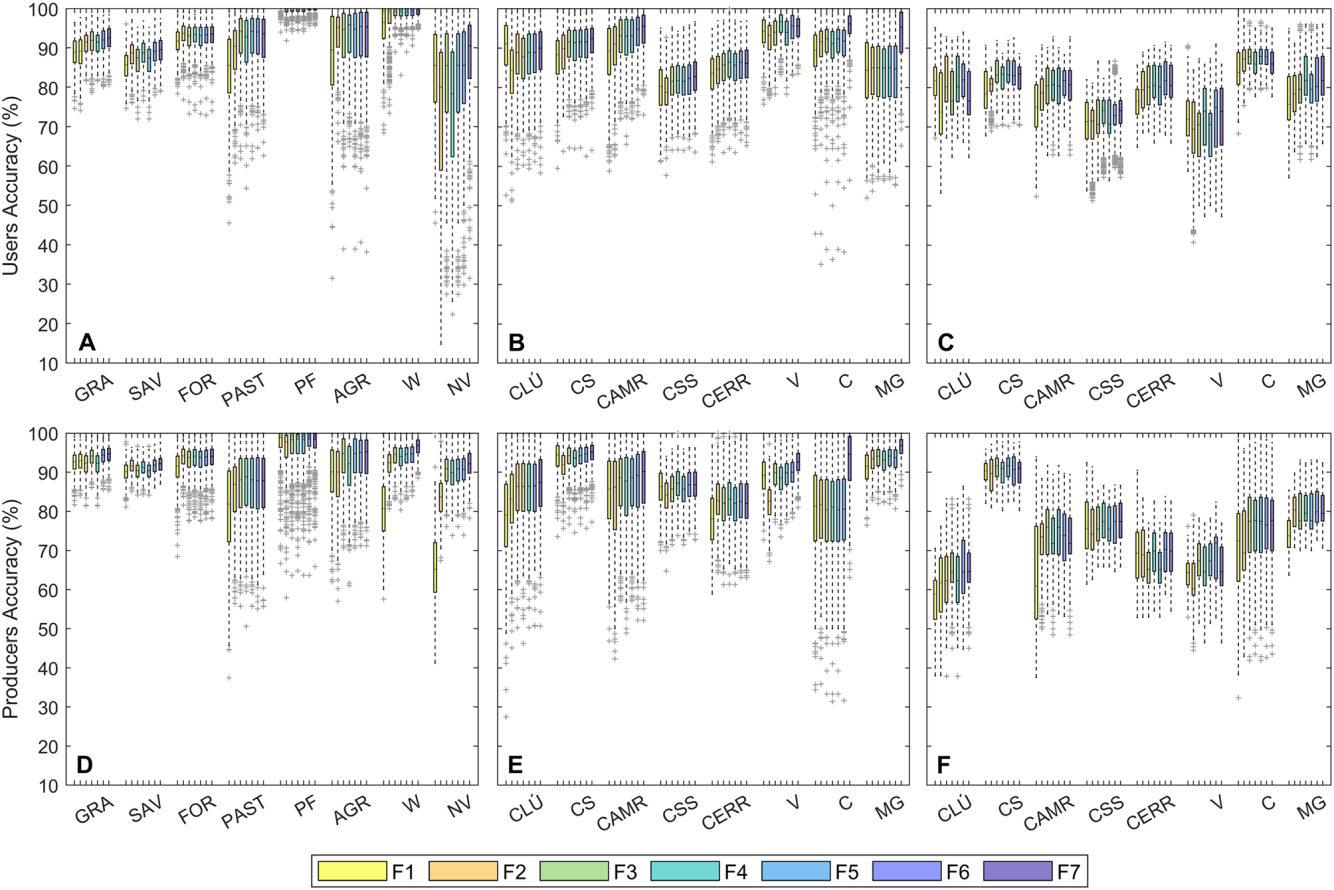
Users and producers accuracies at classification stages 1 and 2. (**A** and **D)** Stage-1 formation level map UA (**A**) and PA (**D**), for each native vegetation, non-native vegetation and non-vegetated class (grass (GRA), savanna (SAV), forest (FOR), pasture (PAST), plantation forest (PF), agriculture (A), water (W) and non-vegetated (NV)) for the seven alternative feature spaces F1–F7. Stage-2 physiognomy level map UA and PA only **(B and E)** and combined stage-1 and 2 UA and PA **(C and F)** for, for each physiognomy ((*campo limpo úmido* (CLÚ), *campo seco* (CS), *campo rupestres* (CAMR), *cerrado sensu stricto* (CSS), *cerrado rupestre* (CER), *vereda* (V), *cerradão* (C) and *mata de galeria* (MG)), across the seven alternative feature spaces F1–F7. For each feature space, the classification process has been repeated 500 times with different training/validation vegetation reference data splits.

Users and producers accuracies were calculated for each formation and physiognomy level class. For all stage-1 formation level classes produced using each alternative feature space, both UA and PA were above 80% (Fig. 4A, D, Table S3). Monoculture plantation forests had the highest UA and PA of all stage-1 vegetated classes, and savannas (UA) and pastures (PA) the lowest. Across the native vegetation formations, differences in map UA or PA between the alternative feature spaces remained small (2.0 to 3.9%). However, when considering both commission and omission errors, F6 performed significantly better than F1 (L8 derived feature space) across all three native formations (p < 0.001). UAs and PAs were significantly lower for non-SAR (F3) feature spaces when compared to F6 for herbaceous and savanna formations, with no significant change for forest formations (p < 0.05). The seasonal feature space (F6) performed better than the annual feature space (F2) for both savanna and grassland formations (UA, p < 0.05). The inclusion of BA masking (F5, F6) significantly improved UA and PA for savanna and grassland formations (p < 0.05). Median UAs range from 85.6% to 93.6%, and PAs from 90.2% to 94.7%, with the lowest UA and PA observed for savanna formations across all feature spaces compared.

At the physiognomy level, UA and PAs at stage-2 only are presented in Fig. 4B and E, combined stage-1 and stage-2 classification errors are also presented in Fig. 4C and F. All further physiognomy level results report the combined stage-1 and stage-2 classification errors (Fig. 4C, F, Table S4). Compared to OAs, UA and PAs were more variable when comparing different classes and input feature spaces, however per class inter-feature space differences in map accuracy remained small. Considering only feature spaces which result in the highest OAs at the second classification stage (F7 and F6, seasonal S1 and S2 with and without texture layers successively), both UA and PA were well above 70% for the majority of physiognomies (Table S4). With the exception of *cerradão* (UA) and *cerrado rupestre* (PA), both commission and omission errors significantly increased (p < 0.05) across all classes when annual feature imagery (F2) was used in place of an equivalent seasonal feature space (F6). For grassland physiognomies, optimised UAs (the highest per class accuracy across all feature spaces assessed) were high for all physiognomies (81.8–83.4%), however the optimised PA was low for *campo úmido* (64.6%) (Fig. 4C, F, Table S3). Across the savanna physiognomies UA and PAs for *cerrado *sensu stricto*, cerrado rupestre* and *veredas* ranged from 60.2 to 81.8 (Table S3). Forest physiognomies: *cerradão* and *mata de galeria* were the most accurately mapped at stage-2. Including texture layers (F7) appeared to improve classification UA and PA for both forested classes when compared to the equivalent non-texture feature space at the second stage (F6) (Fig. 4B, E).

### CVNP landcover maps and probability

**Figure 5:**
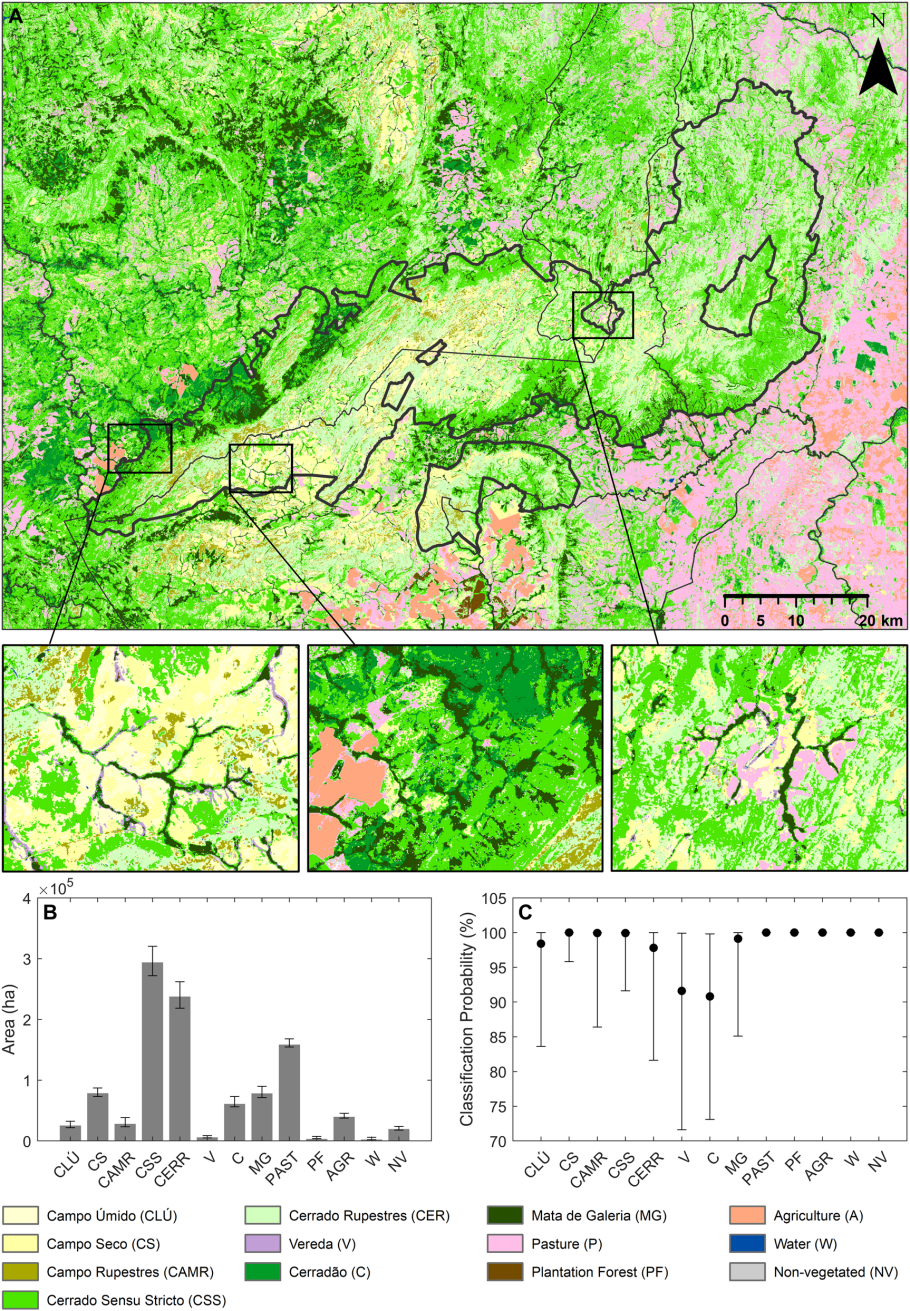
Landcover in the CVNP region. (**A)** Physiognomy level land cover map across the study area, produced using feature space F6, majority classification after 500 classification process repeats. (**B)** Estimated area (ha) of each landcover class across the study region following Olofsson et al. (2013). The median area and IQR of the 95% CI considering all 500 unique classification outputs is indicated. (**C)** Per pixel classification probabilities for each physiognomy (median and IQR, per class, across the study site, including only pixels where a single class majority (> 50%) was reached).

Although it resulted in the second highest map OA at stages 1 and 2 when comparing the seven alternative input feature spaces, the classification map presented here was produced using feature space F6 (Fig. 5A). Incorporating texture imagery (F7) improved map OA at both classification stages, however, it also lead to misclassifications at land cover transitions and at the edges of some physiognomies in areas where native vegetation is more highly fragmented (Fig. S9). As a result, the accuracies reported for F7 are likely to be artificially high, due to the placement of ground reference data within areas of homogenous vegetation, avoiding these transitional areas, and not reliably representing them in map accuracy metrics^98^.

After classification stage-1, a majority formation level class, where a single class was output for > 50% of the 500 classification runs with unique ground reference training subsets, could be assigned for 99.5% of pixels across the study site. At the second classification stage, 97.9% of pixels classified as grassland, 98.6% for savanna and 97.7% for forest at stage-1 could be classified at the physiognomy level. *Cerrado *sensu stricto was the most common native vegetation class across the study area, followed by *cerrado rupestre*, with *vereda*s the rarest class (Fig. 5B). Median per pixel classification probabilities for each native physiognomy ranged from 90.8% (IQR = 26.7%) to 100% (IQR = 0.1%) (including only pixels where a majority has been reached, Fig. 5C).

## Discussion

### Capacity to map Cerrado vegetation at the formation and physiognomy levels

We demonstrate that freely available remote sensing imagery, and a Random Forest classifier implemented in GEE, are sufficient to separate all non-native and most native vegetative Cerrado land covers considered here. This is true at both the formation and finer physiognomy levels using our approach. At the initial stage, native and non-native vegetation covers were easily distinguishable, this is reassuring, particularly in the case of separating native grassland vegetation and high biomass exotic pasture species. This separation is critical for reliably detecting the majority of land cover conversions to pasture^34^, identifying potential restoration area^40^ and assessing the risk of invasion of exotic grassy species for early-stage restoration projects^53^. Within this study we used, high biomass, highly managed pastures, dominated by planted exotic species^99^, as our ground reference dataset. However, pastureland definitions, management strategies and levels of degradation can vary widely^100,101^. These variations are likely to impact our ability to reliably separate pastures from native grassland physiognomies at wider scales, and perhaps within our study area, especially when pastures consist of a mixture of native and non-native grass species. At the formation level, accuracies when differentiating grassland, savanna and forest formations were higher than most biome scale map products across the Cerrado^34,36^. This may be because these ecoregion scale studies rely on a far lower density of ground reference data across a larger area than used here. At the physiognomy level accuracies were comparable to, or an improvement over other case studies where freely available satellite imagery has been used^66,74^. However, our method offers a key advantage in such that it can easily be scaled-up to a much wider study area, without auxiliary empirical environmental data, and using imagery from a single annual period.

Users and producers accuracies were high for most grassland physiognomies (Fig. 4C, F, Table S4). Two grassland physiognomies; the *campo limpo seco* and *campo sujo* classes are mapped as a single class here (*campo seco*). Both are well-drained, with a high species overlap in the herbaceous-shrub stratum^56^ and can be difficult to identify in the field at fine spatial scales^16^. Other grassland physiognomies; *campo limpo úmido* and *campo rupestre* were reliably detectable in this study. *Campo limpo úmido* has near annually waterlogged soils and is floristically distinct when compared to *campo seco* vegetation^55,56,59^. *Campo limpo úmido* is likely to have a very high soil organic matter content, relative to other grassland and woody physiognomies^7,102,103^. The ability to detect remaining *campo limpo úmido* and protect it is therefore valuable from a conservation and a climate change mitigation perspective. Similarly, *campo rupestre* vegetation is highly biodiverse with extremely high numbers of endemics and a high functional diversity in order to tolerate different environmental stressors, however, the area of *campo rupestre* across Brazil is low^104,105^, thus making accurate detection highly valuable to conservation efforts.

Within the savanna formation, *veredas* had the lowest UA within our study. Due to the low density of palms and their distinct linear positioning or tight clustering, usually following drainage lines, the riparian zone of streams or depressions in the landscape^93,106^, reliably detecting *veredas* using 20 to 30 m spatial resolution imagery remains challenging. Other savanna formations were however, easily distinguishable. *Cerrado rupestre* was separable from *cerrado *sensu stricto*,* perhaps due to differences in relief, geology, density of woody species, extent of exposed surface and soil characteristics, despite woody species composition showing some similarities^107,108^. These two physiognomies have also proven to be separable using multispectral optical satellite imagery in previous case studies, including those carried out in the CVNP region^109^.

The two forest formations, *mata de galeria* and *cerradão*, had a consistently high UA and PA within our study. *Mata de galeria* woody species are floristically distinct, often semi-deciduous or with a low Leaf Mass per Area (LMA) starkly contrasting *cerradão*^33,110^ and most other common woody Cerrado species. *Cerradão* was also very distinct from *cerrado *sensu stricto, despite the high number of woody species these two physiognomies share^33^. Often the only differentiating factor between these two physiognomies is increased woody vegetation cover and height, and lack of herbaceous cover in *cerradão*, likely indicative of slight elevations in soil fertility and lower frequency fires^33,111^. Critically, both forest physiognomies have high standing biomass stocks^112,113^, and are important for landscape connectivity^114^, contributing to their importance from a conservation and restoration perspective. Overall, the majority of classes could be separated at the physiognomy level using this approach, highlighting the heterogeneity of Cerrado vegetation at the landscape scale, however our capacity to achieve this separation varied according to the input imagery feature space used.

### Impacts of varying classification input imagery

Although altering the combination of imagery used in the classification process did result in significant changes to map OA, UA and PA at the formation and physiognomy level, the resulting differences in map accuracies observed here were surprisingly small. Optical vegetation indices (EVI2, NDVI) are important for identifying vegetation cover in landscapes that span grassland-savanna-forest transitions^111^ (Fig. S2B). There were some marginal benefits of using an equivalent Sentinel-2 over Landsat-8 derived feature space in terms of accuracy in addition to the benefits of a finer final map spatial resolution when using Sentinel-2 (Table 4). These advantages were more evident at the physiognomy level (Fig. 3B). This improvement in accuracy may be attributed to the addition of red-edge bands in the S2 product, which provide further information on vegetation reflectance at wavelengths between the Red an NIR bands, where spectral reflectance signatures for vegetation typically change rapidly (Table 3, Fig. S2A)^115^. The decrease in accuracy observed when using L8 may also be due to differences in imagery spatial resolution. Additionally, if this approach was expanded to other tropical areas, the high revisit frequency of S2 when compared to L8 may increase the chance of obtaining cloud free imagery^116^. However, it is encouraging that reasonable accuracies can be produced using L8, given the much longer time series this potentially allows for this type of analysis (similar imagery derived from predecessors to the L8 satellite are available from 1982 onwards, whereas Sentinel-2 data is only available from 2015^86,87^).

Including seasonal imagery and phenological response indicators (annual variance, Fig. S2C and S4B) for certain imagery sets appeared to have the greatest impact on classification accuracy at stage-2 (Fig. 3B). Many studies use simple vegetation phenological metrics, like seasonal imagery, within the classification feature space to differentiate between vegetation types in semi-arid or seasonally dry environments^34,117^. Cerrado physiognomies generally have different phenological responses to drought. Consequently, the inter and intra-physiognomy timing of dry-season die back and wet season-green up varies between physiognomies^66,118,119^, making seasonal imagery a key tool for separating physiognomies in this study. Several studies have used high frequency timeseries imagery to monitor annual variation in vegetation productivity, this allows the quantification of the magnitude and timing of phenological responses to water deficit to be incorporated directly into vegetation classification attempts^66,73,74,120^. However, the application of freely available high frequency timeseries imagery to identify complex phenological metrics (rather than aggregating seasonal data) remains difficult in the Cerrado due to cloud cover^121^ and frequent burn events (Fig. 2).

When compared to the inclusion of seasonal imagery, the benefits of incorporating C-band SAR into our classification feature space were even more marginal. The advantages of using SAR products, both exclusively and in combination with optical imagery for monitoring ecosystem functioning and vegetation mapping have been demonstrated across several studies^83,84,122,123^. Inter-season changes in small scale, top of canopy and leafy vegetation structure, soil and biomass moisture are evident in the grassland physiognomies, particularly in the VH polarisation (Fig. S4)^84,124,125^. However, incorporating C-band SAR only minimally improved the classification above what was possible when using optical imagery alone (Fig. 3). Utilizing L-band SAR, where freely available (for instance the PALSAR-2/PALSAR Mosaics^126^) could potentially improve vegetation type separation capability further, by providing information about woody vegetation structure and biomass gradients^127–129^. Furthermore, the addition of texture imagery resulted in systematic misclassifications of some physiognomies which were not captured in our error metrics, despite appearing to improve classification OA^98^. This is likely due to a lack of reference data located at the edges of transitions between vegetation types.

Disregarding reference data that may have burned over the imagery period marginally improved classification accuracies for the savanna and grassland formations. This quality control step may have prevented bare ground pixels and recovering post-burn vegetation pixels from being included in the training dataset (Fig. 2)^80,130^. The impact of vegetation burning on the collection of reliable ground reference data and the subsequent effect burn events have on classification accuracies will likely vary across the Cerrado, as natural and anthropogenic fire regimes differ^131^. In addition, the accuracy of the global BA products used here, when assessed globally and across the Cerrado was found to be low in multiple studies^80,132,133^ as mirrored in the results of this study for the CVNP region (Fig. S6). Several novel methodologies have recently been developed to improve Cerrado burned area monitoring^134,135^.

Despite some imagery and products appearing more important than others for the separation of different vegetation types, it seems a seasonal, combined Sentinel-2 and Sentinel-1 feature space may have the greatest potential for mapping vegetation across the Cerrado.

### Limitations and further work

The capacity to undertake this methodology using freely accessible imagery within the GEE platform, taking advantage of cloud computing capabilities, means that this methodology is comparatively easy to upscale to a biome wide study region^62^. Despite this, upscaling is still likely to present additional challenges. Across the Cerrado, physiognomy species compositions^14^, vegetation functioning, climate and phenology^33,39,110^, and the level of degradation and fragmentation^34^ can be highly variable. This is particularly pronounced at the transitional zones between the Cerrado and other biomes; Amazonia, Mata Atlantica and the Caatinga^6,136,137^. Therefore, the signature of each physiognomy within each imagery set is likely to change across the Cerrado. A large amount of recently collected ground reference data would likely be needed to produce a map across such a large area, and this is likely to be logistically challenging and time consuming to collect. In this study, although roughly consistent at each stage (Table S1), the number of reference pixels sampled varied between vegetation types, which may adversely impact results^138^. Furthermore, the classification of many of these physiognomies in the field is challenging, categorizing vegetation based on characteristics is often subjective and across the literature their definitions vary often with overlap between classes, a problem that will become more pronounced when working across a wider area^16,54^. Consequently, upscaling will still require a considerable amount of data, collected over different time points with a standardised methodology for classifying the physiognomies. Further work should also aim to include important Cerrado physiognomies that weren’t considered here, for example *mata seca* (Table 1)^139^. Finally, this methodology identifies vegetation at a single point in time and to further aid in understanding ecosystem functioning, and facilitating conservation and restoration efforts, timeseries maps with native vegetation identified at the physiognomy level will be essential^34,50,61^. This methodology may also be valuable for mapping vegetation in other heterogeneous seasonally dry regions across the Tropics^1^.

## Conclusion

In this study, we show that it is possible to detect and map a range of non-native and native Cerrado physiognomies to within a reasonable level of accuracy using freely available imagery. When burned ground reference data masking is undertaken, a combination of seasonal and annual variance imagery, incorporating both surface reflectance and C-band SAR data produced the highest map accuracies across the CVNP region. As this study utilises accessible cloud computing capabilities (GEE) alongside free satellite derived imagery, this methodology may be more easily upscalable than other techniques, if sufficient ground reference data can be collected. If trailed in different locations and extended across wider areas of the Cerrado, this approach may be valuable for improving our understanding of the functioning of native vegetation at the landscape scale and how best to implement conservation and restoration projects.

## Supplementary Information

Supplementary Information.
