# Supplementary material for: Mapping native and non-native vegetation in the Brazilian Cerrado using freely available satellite products
Source: Sci Rep. 2022 Jan 28;12:1588. doi: 10.1038/s41598-022-05332-6 (PMC8799689; doi:10.1038/s41598-022-05332-6)
Supplement: Supplementary file 1 — Supplementary Information. [file 41598_2022_5332_MOESM1_ESM.pdf]

Supplementary Material

Mapping native and non-native vegetation in the Brazilian Cerrado using freely available satellite products

Kennedy Lewis[1*], Fernanda de V. Barros[1], Marcio B. Cure[2], Christian A. Davies[3], Mariana N. Furtado[4], Timothy C. Hill[1], Marina Hirota[5], Demétrius L. Martins[4], Guilherme G. Mazzochini[4], Edward T. A. Mitchard[6], Cássia B. R. Munhoz[7], Rafael S. Oliveira[4], Alexandre B. Sampaio[8], Nicholas A. Saraiva[9], Isabel B. Schmidt[10] and Lucy Rowland[1]

1. *College of Life and Environmental Sciences, University of Exeter, Exeter, UK*
2. *Department of Ecology and Zoology, Universidade Federal de Santa Catarina (UFSC), Florianópolis, Brazil.*
3. *Shell International Exploration and Production Inc. (USA), Shell Technology Centre Houston, Houston, TX, USA*
4. *Department of Plant Biology, Institute of Biology, Universidade Estadual de Campinas (UNICAMP), Campinas, Brazil.*
5. *Department of Physics, Universidade Federal de Santa Catarina (UFSC), Florianópolis, Brazil.*
6. *School of GeoSciences, University of Edinburgh, Edinburgh, UK*
7. *Department of Botany, Universidade de Brasília, Brasília, Brazil*
8. *Centro Nacional de Avaliação da Biodiversidade e de Pesquisa e Conservação do Cerrado CBC, Instituto Chico Mendes de Conservação da Biodiversidade (ICMBio), Universidade de Brasília, Brazil*
9. *Fundação Oswaldo Cruz, Rio de Janeiro, Brazil*
10. *Department of Ecology, Universidade de Brasília, Brasília, Brazil*

**Author for correspondence (kl378@exeter.ac.uk).*

Table S1. Total number of unique vegetation reference zones and pixels sampled after burned area masking for each Cerrado physiognomy and land cover type.

Land Cover Class	Vegetation Reference Zones	Sampled Pixels (post burned area masking)
**Grassland Formations (canopy <5%)**		(2704)
Campo limpo úmido (CLÚ)	104	729
Campo seco (CS)	237	1565
Campo rupestre (CAMR)	90	410
**Savanna Formations (5%> canopy <60%)**		(2404)
Cerrado sensu stricto (CSS)	347	1042
Cerrado rupestre (CERR)	188	921
Vereda (V)	412	441
**Forest and Woodland Formations (canopy <60%)**		(1495)
Cerradão (C)	54	710
Mata galeria (MG)	254	785
**Non-native Formations**		
Plantation Forest (PF)	54	(1275)
Pasture (PAST)	122	(2563)
Agriculture (AGR)	87	(3095)
Water (W)	383	(383)
Non-vegetation (NV)	270	(207)
**Total (native veg)**	1686	6603
**Total**	2629	14190

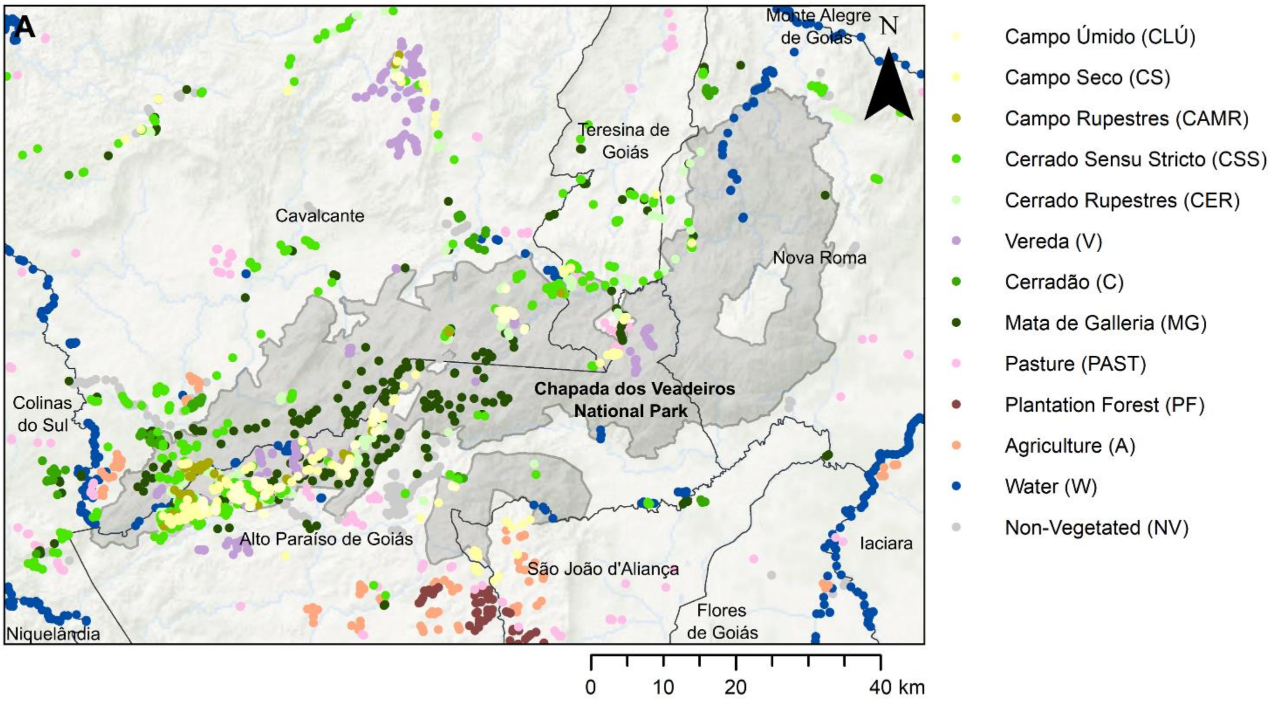

Figure S1. Location of vegetation reference zones across the study area. *To ensure consistency amongst ground truth points and remove data that may be erroneous, reference points were checked after collection in comparison to multiple years of imagery in Google Earth (from 2016 – 2020). In some instances, topographic profiles were also used to assess the reliability of the data collected.*

Table S2. Input feature layers for the alternative feature spaces (F1 – F7). *Input layers for each feature space are indicated including annual (Ann, 01/10/18 to 30/09/19), wet season (Wet, 01/10/2018 - 31/03/2019) and dry season (Dry, 01/04/2019 - 30/09/2019) images, annual pixel variance (A_{var}), wet and dry season texture images (T_W, T_D). Feature imagery is the same in F5 and F6, however classification F5 is run without vegetation reference data burned area masking.*

**Feature Layer**	**Feature Space**						
	**F1**	**F2**	**F3**	**F4**	**F5**	**F6**	**F7**
**Sentinel-1**							
σ^0_{VH}dB Ann		X					
σ^0_{VH}dB Wet	X			X	X	X	X
σ^0_{VH}dB Dry	X			X	X	X	X
σ^0_{VH}dB Avar	X			X	X	X	X
σ^0_{VV}dB Ann		X					
σ^0_{VV}dB Wet	X			X	X	X	X
σ^0_{VV}dB Dry	X			X	X	X	X
σ^0_{VV}dB Avar	X			X	X	X	X
$\sigma^0_{VH_VV}$dB Ann		X					
$\sigma^0_{VH_VV}$dB Wet	X			X	X	X	X
$\sigma^0_{VH_VV}$dB Dry	X			X	X	X	X
**Sentinel-2**							
Blue Ann		X					
Blue Wet			X	X	X	X	X
Blue Dry			X	X	X	X	X
Green Ann		X					
Green Wet			X	X	X	X	X
Green Dry			X	X	X	X	X
Red Ann		X					
Red Wet			X	X	X	X	X
Red Dry			X	X	X	X	X
Red A_{var}			X		X	X	X
Red Edge 1 Ann		X					
Red Edge 1 Wet			X	X	X	X	X
Red Edge 1 Dry			X	X	X	X	X
Red Edge 1 A_{var}			X		X	X	X
Red Edge 2 Ann		X					
Red Edge 2 Wet			X	X	X	X	X
Red Edge 2 Dry			X	X	X	X	X
Red Edge 2 A_{var}			X		X	X	X
Red Edge 3 Ann		X					
Red Edge 3 Wet			X	X	X	X	X
Red Edge 3 Dry			X	X	X	X	X
Red Edge 3 A_{var}			X		X	X	X
NIR Ann		X					
NIR Wet			X	X	X	X	X
NIR Dry			X	X	X	X	X
NIR A_{var}			X	X	X	X	X
Red Edge 4 Ann		X					
Red Edge 4 Wet			X	X	X	X	X
Red Edge 4 Dry			X	X	X	X	X
Red Edge 4 A_{var}			X		X	X	X

**Sentinel-2 (Continued…)**							
SWIR 1 Ann		X					
SWIR 1 Wet			X	X	X	X	X
SWIR 1 Dry			X	X	X	X	X
SWIR 2 Ann		X					
SWIR 2 Wet			X	X	X	X	X
SWIR 2 Dry			X	X	X	X	X
NDVI Ann		X					
NDVI Wet			X	X	X	X	X
NDVI Dry			X	X	X	X	X
NDVI A_{var}			X		X	X	X
NDVI T_W							X
NDVI T_D							X
EVI2 Ann		X					
EVI2 Wet			X	X	X	X	X
EVI2 Dry			X	X	X	X	X
EVI2 A_{var}			X		X	X	X
EVI2 T_W							X
EVI2 T_D							X
SAVI Ann		X					
SAVI Wet			X	X	X	X	X
SAVI Dry			X	X	X	X	X
SAVI A_{var}			X		X	X	X
SAVI T_W							X
SAVI T_D							X
SWIR21 Ann		X					
SWIR21 Wet			X	X	X	X	X
SWIR21 Dry			X	X	X	X	X
SWIR21 A_{var}			X		X	X	X
**Landsat-8**							
Blue Wet	X						
Blue Dry	X						
Green Wet	X						
Green Dry	X						
Red Wet	X						
Red Dry	X						
Red A_{var}	X						
NIR Wet	X						
NIR Dry	X						
NIR A_{var}	X						
SWIR 1 Wet	X						
SWIR 1 Dry	X						
SWIR 1 Wet	X						
SWIR 1 Dry	X						
NDVI Wet	X						
NDVI Dry	X						
NDVI A_{var}	X						
EVI2 Wet	X						
EVI2 Dry	X						
EVI2 A_{var}	X						
SAVI Wet	X						
SAVI Dry	X						

**Landsat-8 (Continued…)**							
SAVI A_{var}	X						
SWIR21 Wet	X						
SWIR21 Dry	X						
SWIR21 A_{var}	X						
**COP-DEM GLO-30**							
Slope	X	X	X	X	X	X	X

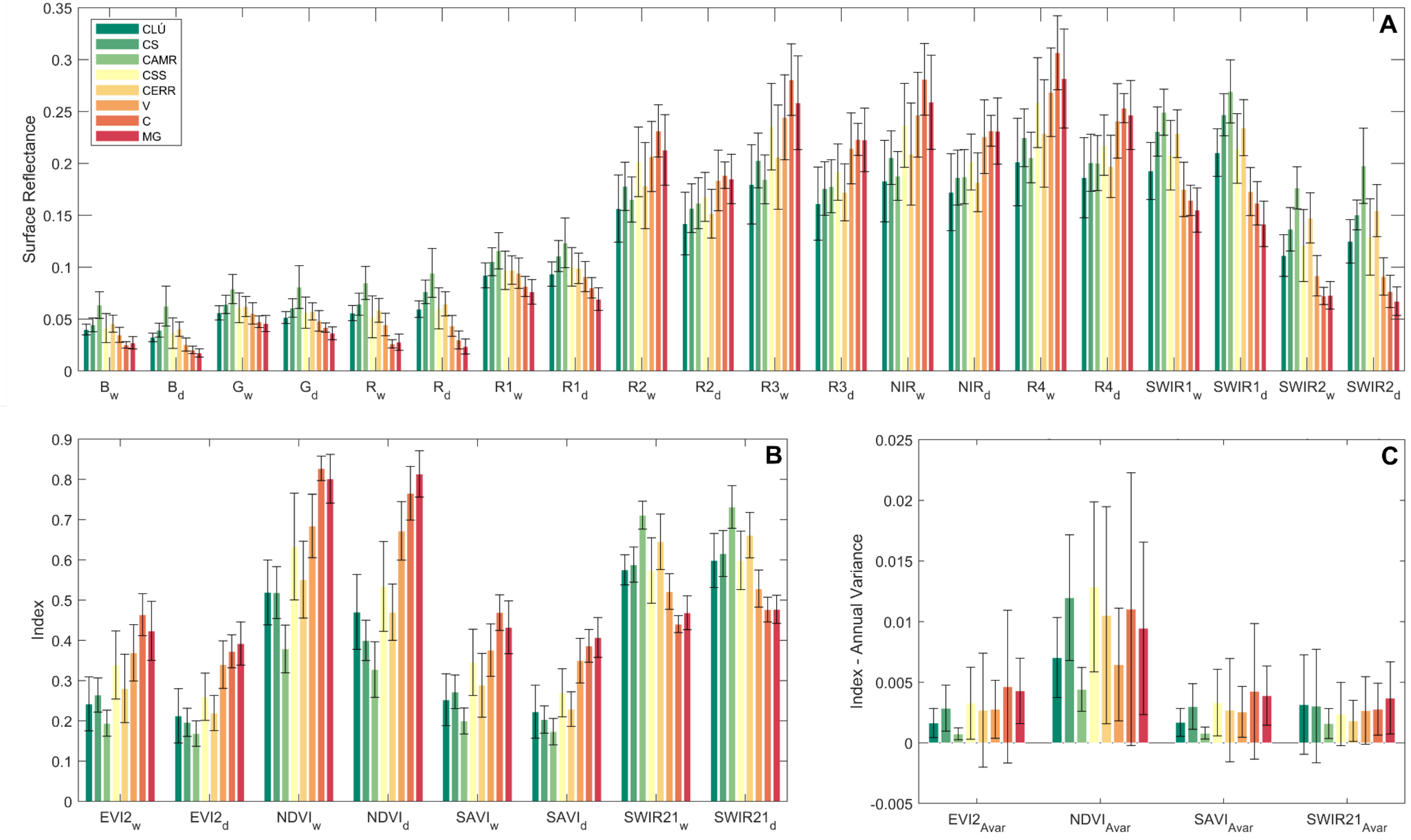

Figure S2. Sentinel-2 signatures for native Cerrado physiognomic classes. *Mean seasonal signatures for the S2 feature layers across all ground reference pixels for each class, standard deviation indicated, see Table 2 for layer names.* ***A.*** *Seasonal S2 surface reflectance for each available band.* ***B.*** *Seasonal vegetation indices.* ***C.*** *Annual per pixel variance of vegetation indices across all available cloud free imagery.*

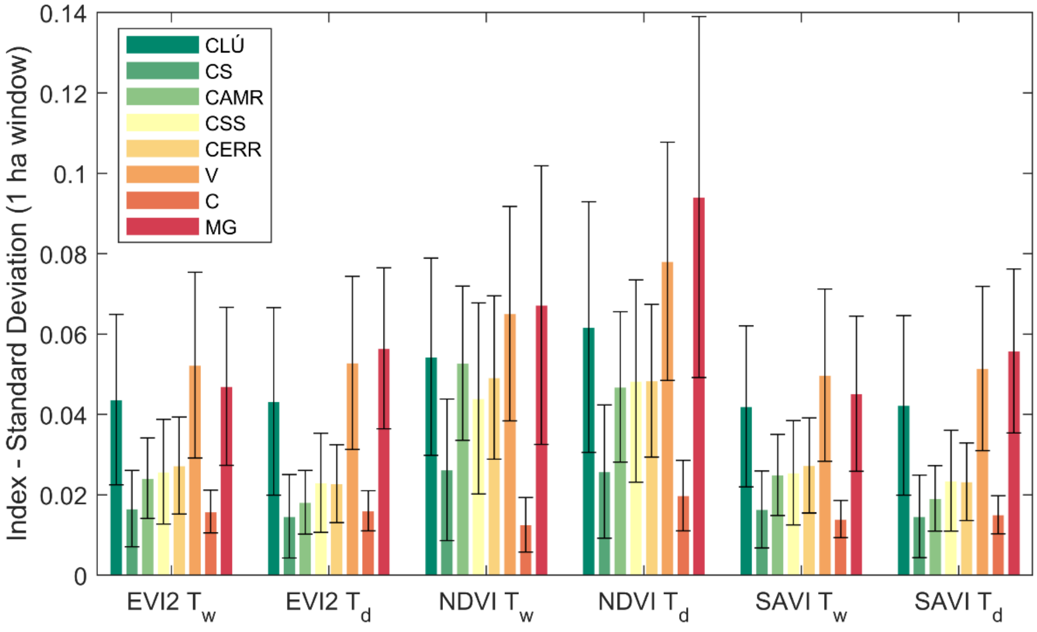

Figure S3. Sentinel-2 texture signatures for native Cerrado physiognomic classes. *Mean seasonal signatures for the S2 vegetation index texture layers (1 ha window), across all ground reference pixels for each class, standard deviation indicated, see Table 2 for layer names.*

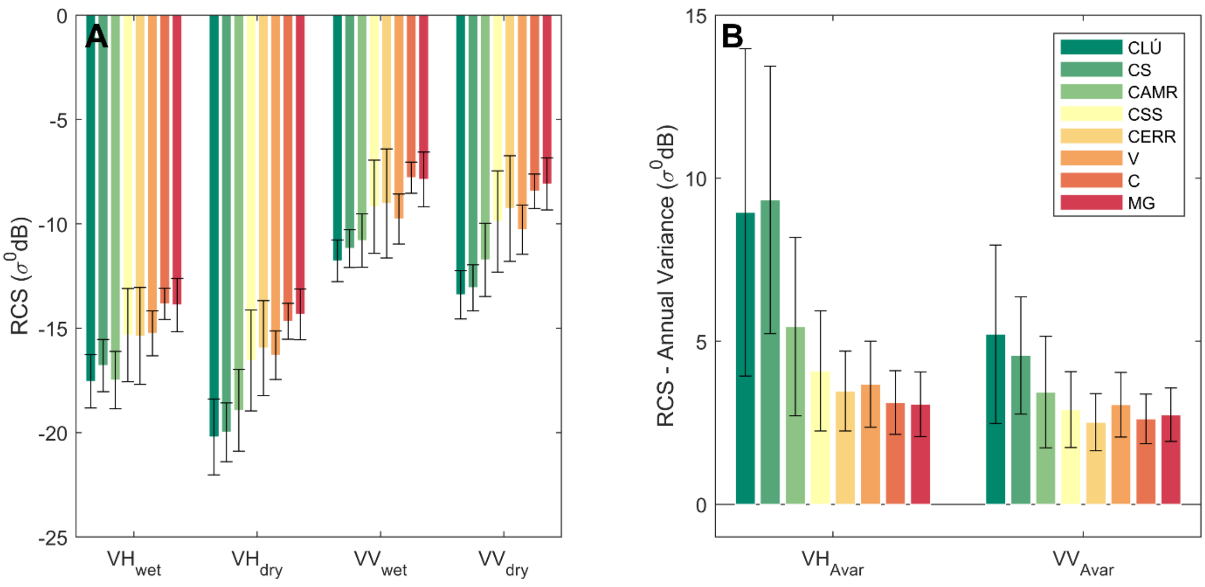

Figure S4. Sentinel-1 signatures for native Cerrado physiognomic classes. *Mean seasonal signatures for the S1 SAR layers at the VH and VV polarisation, across all ground reference pixels for each class, standard deviation indicated, see Table 2 for layer names.* ***A.*** *Seasonal S1 Radar Cross Section (RCS) for each available polarisation.* ***B.*** *Annual per pixel RCS variance across all available imagery.*

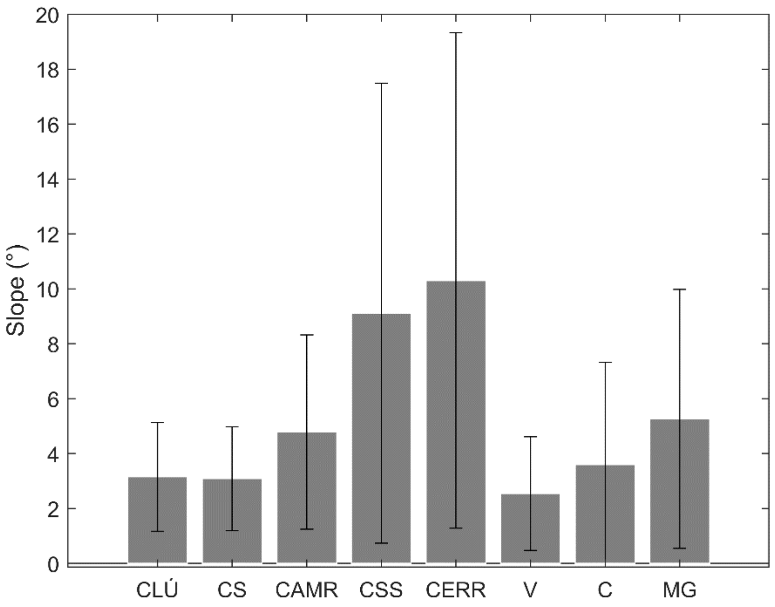

Figure S5. COP-DEM GLO-30 derived slope for native Cerrado physiognomic classes.

Supplementary Section 1

Global burned area product error assessment

The accuracy of the MCD64A1 v6 (resolution 500 m) and ESA Fire_CCIv5 (resolution 250 m) global burned area products was assessed at the study site. Both have a 1 day repeat pass. For the years 2017 to 2019, the burned area from these global products was compared to the burned area taken from the AQ30m maps. AQ30m is a derived from Landsat imagery, specifically for the Cerrado region and has a ground resolution 30 m (Melchiori et al, 2014). The methodology of Rodrigues et al, 2019 was followed.

Available burn scar reference data (AQ30m) was acquired for dry season months (2017 - 2019) where source Landsat scenes had < 10% cloud cover. Visual assessment for clouds/shadows near detected burn scars was undertaken. The mean burn date uncertainty in the MCD64A1 v6 product global product across the site was 1.96 days (no burn date uncertainty metrics are provided for Fire_CCIv5).

Each available reference burn area scene, extending 16 days, was compared to the corresponding MCD64 v6 and Fire_CCIv5 interval (per pixel, reprojected to WGS_1984). Both global burned area products were resampled to a 30 m resolution. To account for burn date uncertainty, this interval was extended by 2 days at the start and end of the period. Commission errors (the fraction of unburned pixels mistakenly classified as burned) and omission errors (the fraction of burned pixels not detected by the product) were calculated for each interval (n = 17). For each global burned area product to comparisons were made, initially all reference burn scars included and subsequently including only reference product burn scars with an area greater than the native resolution of the burned are product.

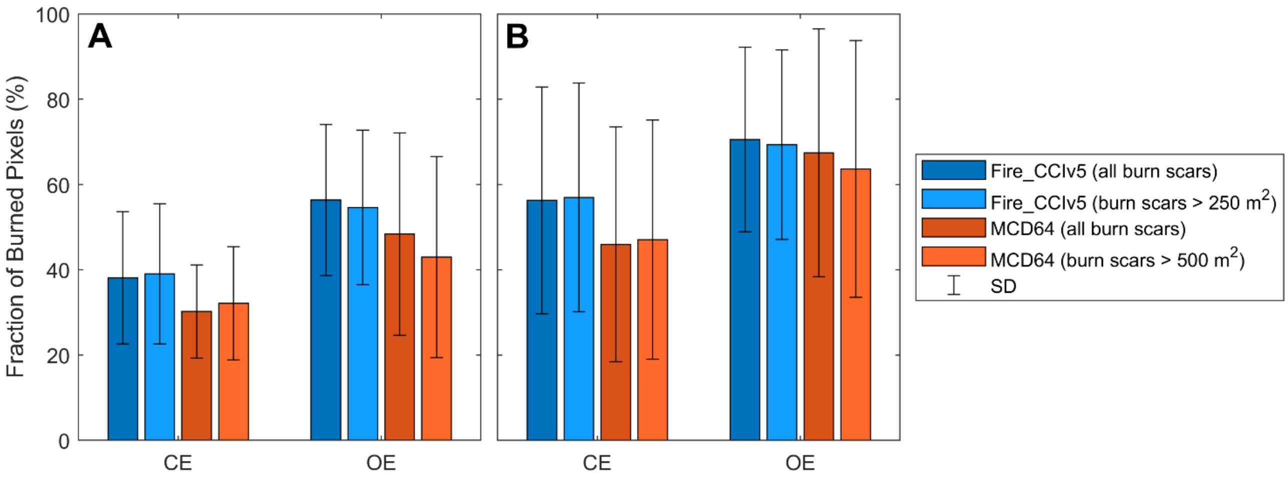

Figure S6. Commission errors (CE) and Omission errors (OE) for reference scene intervals corresponding to the feature imagery period (n = 5, Panel B) and for all available reference scene intervals (2017 - 2019, n = 17, Panel A).

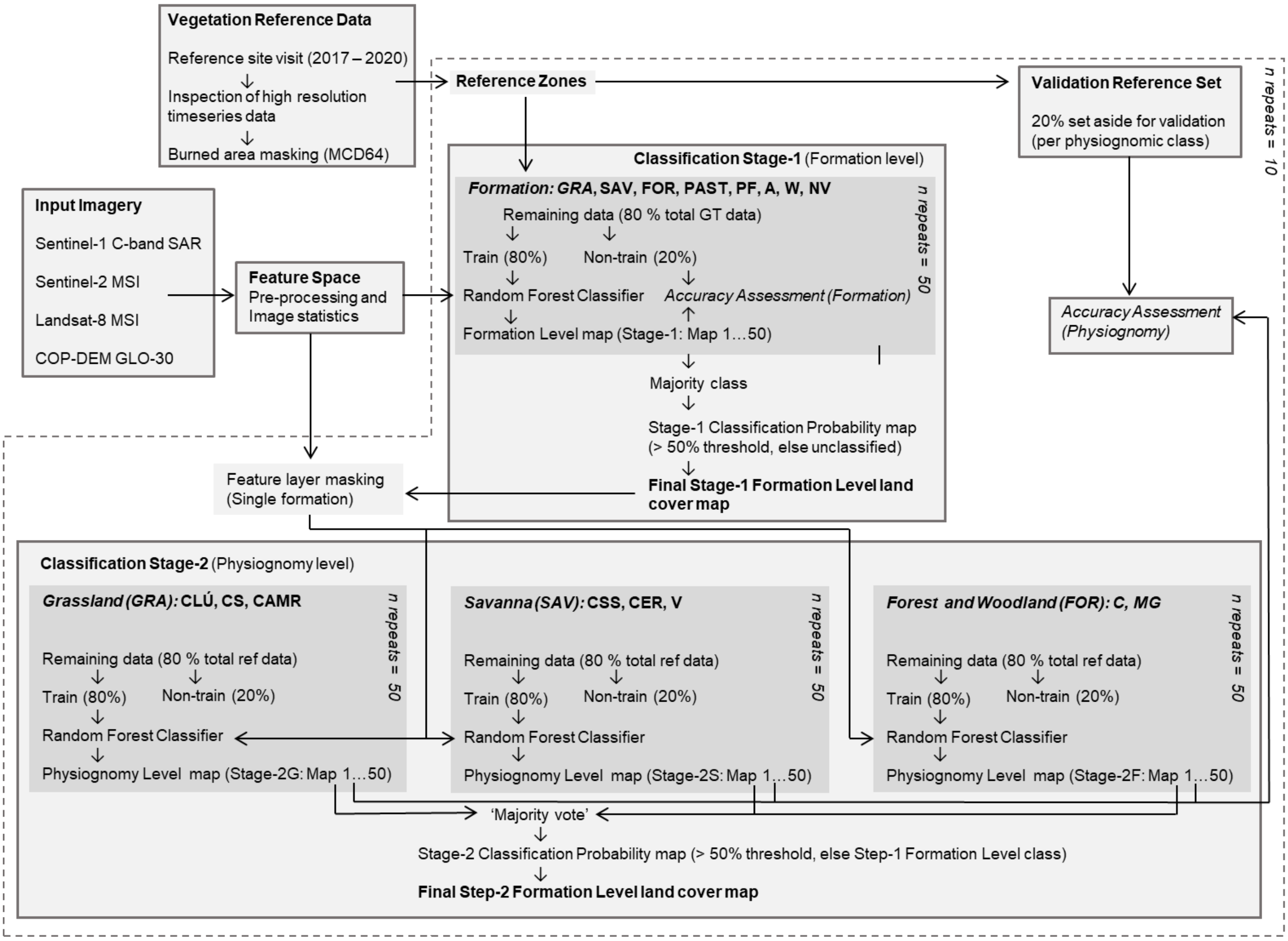

Figure S7. Methodological steps and classification routine. *Formation level classes: Grassland (GRA), Savanna (SAV), Forest (FOR), Pasture (PAST), Plantation Forest (PF), Agriculture (A), Water (W), Non-vegetated (NV). For physiognomy level classes, see Table 1.*

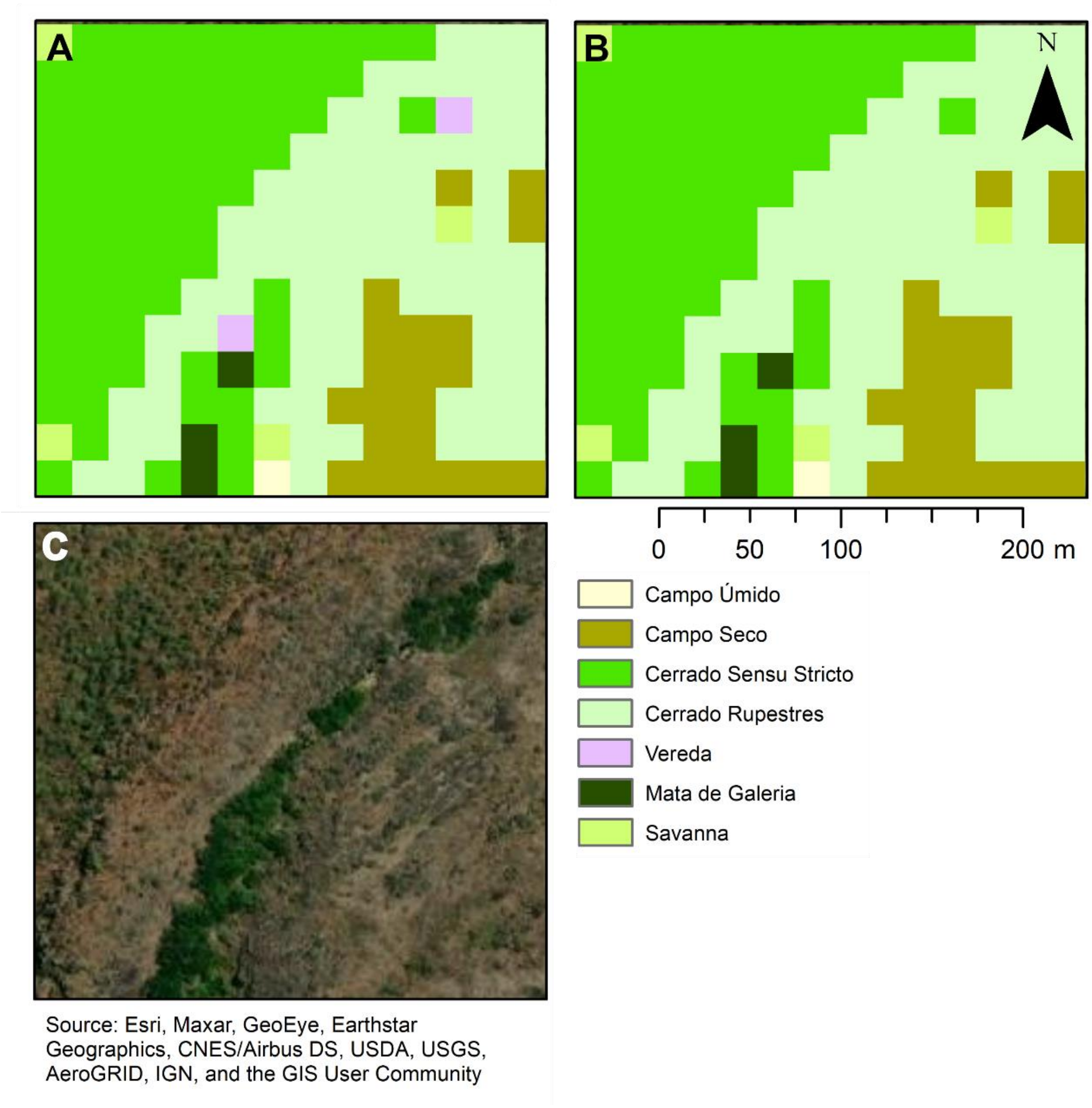

Figure S8. Landcover maps across an area of the CVNP limits produced using F6. A. *Landcover map produced using F6 before distance-based reclassification of vereda pixels (vereda – campo úmido proximity).* ***B.*** *Landcover map produced using F6 after distance-based reclassification of vereda pixels (final landcover map).* ***C.*** *Esri high resolution imagery (see source), no veredas are present across the section presented.*

Table S3. Users and producers accuracies at classification stage 1 (Formation Level). *The median error and interquartile range (IQR) after 500 independent repeats of the classification process are reported for each alternative feature space.*

	**GRA**	**SAV**	**FOR**	**PAST**	**PF**	**AGR**	**W**	**NV**
**Users Accuracy (median (IQR))**								
**F1**	88.8 (5.4)	85.6 (5.2)	91.7 (4.5)	85.5 (13.6)	100.0 (0.0)	89.4 (17.4)	96.4 (7.7)	85.4 (17.1)
**F2**	89.1 (6.1)	88.7 (4.8)	93.6 (3.7)	90.4 (9.8)	99.7 (0.9)	95.2 (7.6)	100.0 (3.8)	80.0 (29.9)
**F3**	91.4 (4.1)	87.4 (5.6)	93.0 (4.6)	94.1 (8.8)	100.0 (0.8)	94.6 (11.2)	100.0 (1.9)	85.5 (19.9)
**F4**	91.9 (4.7)	88.7 (4.8)	93.4 (3.7)	92.8 (10.6)	100.0 (0.8)	95.9 (9.4)	100.0 (1.9)	78.3 (26.6)
**F5**	91.4 (4.1)	87.4 (5.6)	93.0 (4.6)	94.1 (8.8)	100.0 (0.8)	94.6 (11.2)	100.0 (1.9)	85.5 (19.9)
**F6**	91.9 (4.5)	89.1 (4.7)	93.2 (3.8)	94.0 (9.0)	100.0 (0.4)	95.5 (11.1)	100.0 (1.8)	85.6 (18.2)
**F7**	92.4 (4.5)	89.5 (4.9)	93.4 (3.9)	93.5 (9.7)	100.0 (0.5)	95.2 (11.4)	100.0 (1.6)	90.5 (13.5)
**Producers Accuracy (median (IQR))**								
**F1**	92.6 (3.6)	90.2 (3.3)	91.6 (5.3)	81.8 (18.1)	98.9 (3.7)	90.1 (10.7)	80.7 (11.2)	65.2 (12.7)
**F2**	93.0 (3.5)	91.6 (2.7)	94.2 (3.7)	86.9 (11.4)	97.8 (5.7)	90.1 (11.5)	92.5 (4.5)	83.7 (7.1)
**F3**	92.4 (4.1)	90.5 (2.9)	93.8 (4.1)	88.1 (12.6)	98.3 (5.2)	94.8 (8.7)	94.5 (3.8)	90.9 (5.6)
**F4**	94.2 (3.2)	91.2 (2.8)	94.1 (4.0)	88.8 (11.9)	98.5 (4.9)	92.1 (10.0)	94.0 (4.5)	89.9 (6.2)
**F5**	92.4 (4.1)	90.5 (2.9)	93.8 (4.0)	88.1 (12.6)	98.3 (5.2)	94.8 (8.7)	94.5 (3.8)	90.9 (5.6)
**F6**	94.4 (3.1)	91.8 (2.9)	93.9 (3.9)	87.8 (13.0)	98.5 (3.4)	95.0 (8.3)	94.7 (4.0)	90.7 (5.4)
**F7**	94.7 (3.0	92.2 (2.9)	94.0 (3.9)	87.9 (12.6)	98.4 (3.8)	95.2 (8.6)	96.8 (3.2)	92.2 (5.1)

Table S4. Users and producers accuracies at classification stage 2 (Physiognomy Level*). The median error and interquartile range (IQR) after 500 independent repeats of the classification process are reported for each alternative feature space.*

	**CLÚ**	**CS**	**CAMR**	**CSS**	**CERR**	**V**	**C**	**MG**
**Users Accuracy (median (IQR))**								
**F1**	81.7 (7.2)	78.4 (9.3)	75.0 (10.7)	71.4 (9.3)	76.6 (6.3)	72.0 (8.9)	83.8 (8.1)	75.8 (11.0)
**F2**	73.8 (15.5)	80.9 (3.2)	78.6 (7.8)	71.5 (7.3)	79.4 (9.2)	69.4 (13.0)	87.2 (5.4)	78.4 (9.1)
**F3**	80.2 (11.6)	83.2 (5.5)	80.4 (9.0)	70.7 (8.5)	80.2 (9.8)	70.6 (11.0)	87.9 (3.7)	79.5 (7.2)
**F4**	76.6 (11.0)	83.4 (5.6)	80.7 (7.6)	74.2 (5.8)	80.5 (8.2)	74.0 (14.4)	86.0 (5.4)	81.8 (9.8)
**F5**	80.2 (11.6)	83.2 (5.5)	80.4 (9.0)	70.7 (8.5)	80.2 (9.8)	70.6 (11.0)	87.9 (3.7)	79.5 (7.2)
**F6**	82.0 (8.3)	83.0 (6.1)	81.8 (6.2)	72.9 (5.1)	81.8 (8.4)	75.0 (14.4)	87.8 (3.8)	80.2 (11.0)
**F7**	76.6 (11.0)	83.4 (5.6)	80.7 (7.6)	74.2 (5.8)	80.5 (8.2)	74.0 (14.4)	86.0 (5.4)	81.8 (9.8)
**Producers Accuracy (median (IQR))**								
**F1**	58.8 (10.0)	90.3 (4.2)	60.9 (23.7)	75.7 (11.9)	69.3 (11.7)	64.4 (5.4)	72.5 (17.3)	73.8 (6.9)
**F2**	61.7 (13.9)	89.7 (8.0)	73.5 (7.8)	74.2 (10.3)	68.9 (12.0)	60.2 (8.1)	69.3 (15.1)	80.3 (7.5)
**F3**	62.3 (11.8)	91.7 (4.7)	75.9 (11.4)	75.5 (8.7)	65.1 (7.9)	67.3 (8.2)	77.5 (14.1)	77.7 (9.0)
**F4**	64.6 (7.5)	91.0 (5.2)	71.8 (9.1)	77.4 (8.8)	69.9 (9.8)	64.6 (9.9)	77.7 (12.9)	79.5 (6.5)
**F5**	62.3 (11.8)	91.7 (4.7)	75.9 (11.4)	75.5 (8.7)	65.1 (7.9)	67.3 (8.2)	77.5 (14.1)	77.7 (9.0)
**F6**	64.5 (13.6)	90.7 (7.1)	73.8 (12.0)	77.4 (9.6)	70.3 (9.9)	71.9 (10.7)	76.5 (13.0)	79.9 (7.9)
**F7**	64.6 (7.5)	91.0 (5.2)	71.8 (9.1)	77.4 (8.8)	69.9 (9.8)	64.6 (9.9)	77.7 (12.9)	79.5 (6.5)

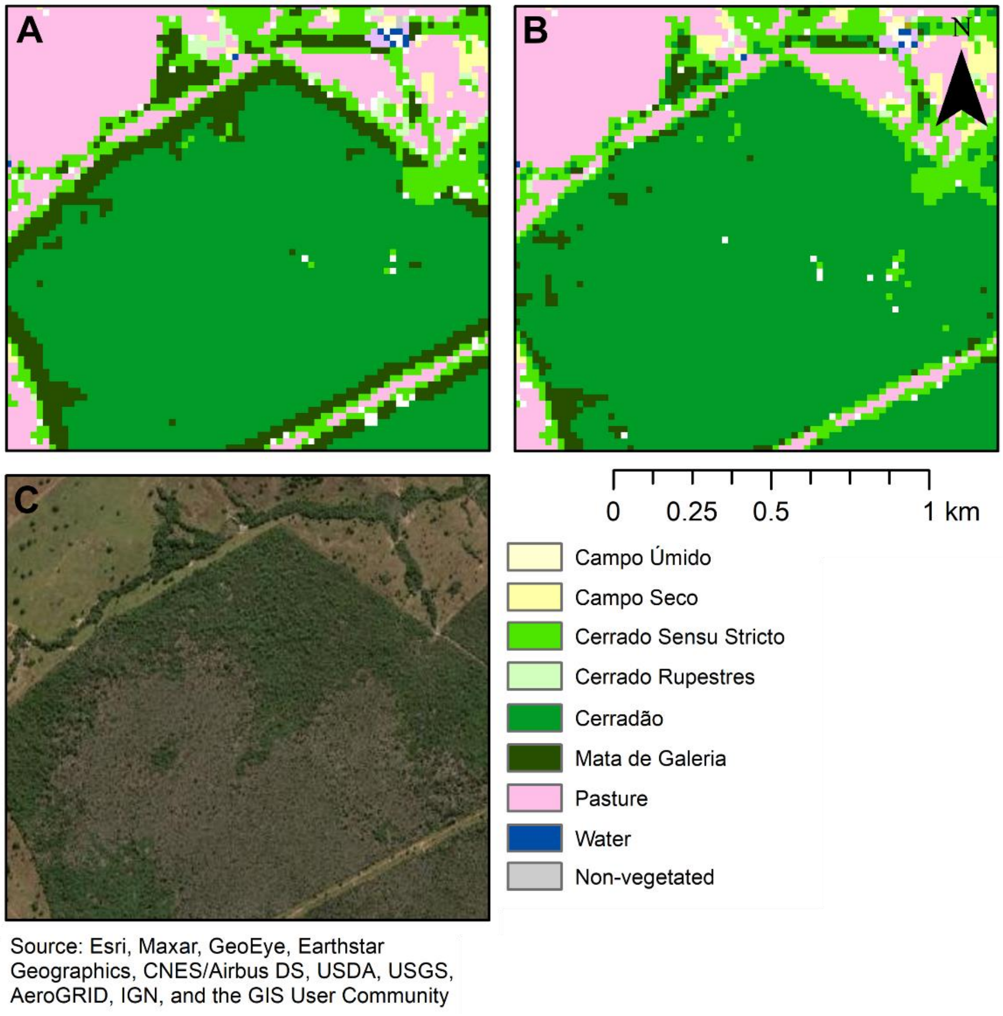

Figure S9. Landcover maps across a cerradão fragment outside of the CVNP limits produced using F6 and F7 (with texture imagery). A. *Landcover map produced using F6.* ***B.*** *Landcover map produced using F7, the mata de galeria class assigned to pixels at the edges of the cerradão fragment.* ***C.*** *Esri high resolution imagery (see source).*

References

1. A.E. Melchiori, A.W. Setzer, F. Morelli, R. Libonati, P. Cândido, A. de & S.C. de Jesus. A Landsat-TM/OLI algorithm for burned areas in the Brazilian Cerrado: preliminary results. *Adv. For. fire Res.* 23-30 (2014).
2. Rodrigues, J. A. et al. How well do global burned area products represent fire patterns in the Brazilian Savannas biome? An accuracy assessment of the MCD64 collections. *Int. J. Appl. Earth. Obs. Geoinf.* **78**, 318–331 (2019).
